# Transcriptome analysis of sugarcane reveals rapid defense response of SES208 to Xanthomonas albilineans in early infection

**DOI:** 10.1186/s12870-023-04073-6

**Published:** 2023-01-24

**Authors:** Yaying Ma, Hongying Yu, Yijing Lu, Sanji Gao, Mahpara Fatima, Ray Ming, Jingjing Yue

**Affiliations:** 1 Center for Genomics and Biotechnology, Fujian Provincial Key Laboratory of Haixia Applied Plant Systems Biology, Key Laboratory of Genetics, Breeding and Multiple Utilization of Crops, Ministry of Education, Fujian Agriculture and Forestry University, Fuzhou, 350002 Fujian China; 2 College of Agriculture, Fujian Agriculture and Forestry University, Fuzhou, 350002 China; 3 Shenzhen Branch, Guangdong Laboratory for Lingnan Modern Agriculture, Genome Analysis Laboratory of the Ministry of Agriculture, Agricultural Genomics Institute at Shenzhen, Chinese Academy of Agricultural Sciences, Shenzhen, 518120 China; 4 National Engineering Research Center for Sugarcane, Fujian Agriculture and Forestry University, Fuzhou, 350002 China

**Keywords:** *Saccharum* spp., *Xanthomonas albilineans*, Leaf scald, RNA-Seq, Disease resistance

## Abstract

**Background:**

Diseases are the major factor affecting the quality and yield of sugarcane during its growth and development. However, our knowledge about the factors regulating disease responses remain limited. The present study focuses on identifying genes regulating transcriptional mechanisms responsible for resistance to leaf scald caused by *Xanthomonas albilineans* in *S. spontaneum* and *S. officinarum.*

**Results:**

After inoculation of the two sugarcane varieties SES208 (*S. spontaneum*) and LA Purple (*S. officinarum*) with *Xanthomonas albilineans*, SES208 exhibited significantly greater resistance to leaf scald caused by *X. albilineans* than did LA Purple. Using transcriptome analysis, we identified a total of 4323 and 1755 differentially expressed genes (DEGs) in inoculated samples of SES208 and LA Purple, respectively. Significantly, 262 DEGs were specifically identified in SES208 that were enriched for KEGG pathway terms such as plant-pathogen interaction, MAPK signaling pathway, and plant hormone signal transduction. Furthermore, we built a transcriptional regulatory co-expression network that specifically identified 16 and 25 hub genes in SES208 that were enriched for putative functions in plant-pathogen interactions, MAPK signaling, and plant hormone signal transduction. All of these essential genes might be significantly involved in resistance-regulating responses in SES208 after *X. albilineans* inoculation. In addition, we found allele-specific expression in SES208 that was associated with the resistance phenotype of SES208 when infected by *X. albilineans*. After infection with *X. albilineans*, a great number of DEGs associated with the KEGG pathways ‘phenylpropanoid biosynthesis’ and ‘flavonoid biosynthesis’ exhibited significant expression changes in SES208 compared to LA Purple that might contribute to superior leaf scald resistance in SES208.

**Conclusions:**

We provided the first systematical transcriptome map that the higher resistance of SES208 is associated with and elicited by the rapid activation of multiple clusters of defense response genes after infection by *X. albilineans* and not merely due to changes in the expression of genes generically associated with stress resistance. These results will serve as the foundation for further understanding of the molecular mechanisms of resistance against *X. albilineans* in *S. spontaneum*.

**Supplementary Information:**

The online version contains supplementary material available at 10.1186/s12870-023-04073-6.

Very high yields of sucrose make *Saccharum officinarum* and other sugarcane species among the most important crops as the largest sources of sugar and biofuel feedstocks from warm climates around the world [1, 2]. In the past few decades, sugarcane has been used not only to generate sugars for food but also to develop potentially sustainable processes to shift the global energy mix toward cleaner and renewable resources [3]. Current cultivated sugarcane hybrids were derived from interspecific crosses between several parental species including *S. barberi*, *S. sinense*, *S. officinarum*, and wild accessions of *S. spontaneum* and *S. robustum* that resulted in complex autopolyploids with 2n = 100–130 chromosomes [4, 5]. The growth and productivity of sugarcane can be very adversely impacted by biotic stress caused by microorganisms and also by abiotic stresses [6]. Researchers have taken several decades to breed cultivated varieties for high sucrose yield and disease resistance. In particular, the high sucrose content of modern sugarcane cultivars was derived from *S. officinarum,* which exhibits poor disease resistance, while their disease resistance was derived from its relative *S. spontaneum*, which exhibits low sugar content. *S. spontaneum* is a key contributor of outstanding traits such as tolerance to environmental stress and ratoon capacity and was therefore used during a program of backcrossing to *S. officinarum*, referred to as nobilization [7]. Thus, considering the genetic background of sugarcane, analysis of the mechanisms of its parental responses to pathogenic bacteria has become necessary. We therefore consider the *S. officinarum* cultivar LA Purple and the *S. spontaneum* cultivar SES208 ideal materials for genetic research into high sugar content and disease resistance traits.

As one of the main diseases of sugarcane (*Saccharum* spp. hybrids), leaf scald can cause the death of the entire sugarcane plant. Due to the potential for leaf scald to cause severe economic losses, this disease has severely restricted the cultivation of susceptible sugarcane varieties [8]. Leaf scald was first recognized as a disease caused by an organism in the Proteobacteria, *Xanthomonas albilineans*. Symptoms of infection with this pathogen in sugarcane have allowed the progression of this disease to be classified into three different stages including incubation period, chronic period, and acute period [9]. *X. albilineans* colonizes the plant vascular system and parenchyma cells [10] and leads to the inhibition of chloroplast development and disruption of photosynthesis [11], followed by “scalding” of stalks and stalk death. A hypersensitive response (HR) is induced early during infection with *X. albilineans*. This pathogen-induced HR is thought to reduce infiltration and spread of the pathogen through local cell death at the site of infection [12, 13].

During their evolution, plants have established an immune system against pathogen infection [14]. Plants live in environments that also host some microorganisms that, as pathogens, can adversely affect their growth and development. To avoid infection by pathogens in their environments, plants respond via an innate immune system [15]. Plants first recognize molecules originating from both pathogenic and non-pathogenic microbes and then respond to virulence factors from pathogens. The primary immune response in plants, known as PAMP-triggered immunity (PTI), involves the recognition of conserved microbial features. PTI is initiated by the perception of pathogens at the cell surface and pathogens that cross the host plasma membrane, which are recognized according to their pathogen-associated molecular patterns (PAMPs) [16]. The hypersensitive response (HR) is the hallmark response of effector-triggered immunity, or ETI, which is initiated directly or indirectly when effectors from pathogens are recognized by NLRs, which are receptors containing nucleotide-binding domains and leucine-rich repeats [15]. Crosstalk between PTI and ETI involves mitogen-activated protein kinase (MAPK) signaling [17] and results in massive accumulation of active oxygen within a short time [18]. A substantial influx of Ca^2+^ [19] and the biosynthesis of plant hormones, including salicylic acid (SA), abscisic acid (ABA), ethylene (ET), and jasmonic acid (JA) also occurs [20]. The beta-hydroxy phenolic compound SA is known as a defense-related hormone that mediates systemic acquired resistance (SAR) [13]. In 1979, aspirin (acetyl-SA) was first reported to increase the resistance of a susceptible tobacco line to infection with TMV (tobacco mosaic virus) [21]. Further, JA- and SA-induced systemic resistance (ISR) can reprogram plant induction mechanisms to alleviate stresses [22]. Plant secondary metabolites, which can be expressed constitutively or in response to pathogens, mediate interactions between plants and microbes in particular [23]. Studies conducted on the activities of these metabolites during disease resistance in plants have shown that many metabolites including phenylpropanoids, terpenoid quinones, and flavonoids can potentially combat plant pathogens [23, 24].

Several studies of plants infected with *X.albilineans* have been carried out in species such as rice [25, 26], elephant grass [27], and wheat [28], and shown that the expression of some genes involved in pathways controlling the biosynthesis and activity of ethylene, jasmonic acid, or secondary metabolites respond to inoculation with this pathogen. Although transcripts of more than 5000 genes with altered expression were previously revealed in an analysis comparing the transcriptomes of sugarcane cultivars that are resistant or susceptible to leaf scald after infection with *X. albilineans* [29], the cellular mechanisms of resistance to this pathogen in sugarcane are still unknown. The first global sugarcane proteome dataset revealed that seven candidate genes associated with “photosynthesis”, “glycolytic process”, “glycosylation process”, “plant innate immune system”, “plant cytochrome P450”, and “non-specific lipid transfer proteins” are expressed in response to *X. albilineans* inoculation in SES208 [30]. However, although some infected plants do not display external symptoms, especially under conditions unsuitable for the pathogen, the reasons for such latent progression through infection phases are not known [9]. Therefore, a qualitative PCR assay was established to detect *X. albilineans* infection, exclude the presence of other pathogens, and monitor epidemics and evaluate resistance in the field [31]. Despite such progress, the mechanisms of the molecular responses of modern sugarcane cultivars to infection with *X.albilineans* are still unclear.

To analyze the origins of *X. albilineans* resistance in modern sugarcane varieties from a cross between *S. spontaneum* exhibiting disease resistance and *S. officinarum* exhibiting high sugar and biomass yields, the leaf scald resistance of these two parental species was compared. In the present study, we used RNA-Seq and weighted gene correlation network analysis (WGCNA) to compare the transcriptomes of the representative sugarcane cultivars SES208 (*Saccharum spontaneum,* 2n *=* 64) and LA Purple (*Saccharum officinarum*, 2n = 80) during their genetic responses to infection with *X. albilineans*. Our analysis identified transcripts of DEGs associated with *X. albilineans* resistance that will be of great significance for improving our understanding of *X. albilineans* resistance in sugarcane.

## Results

### Differences in responses of sugarcane cultivars LA purple and SES208 to inoculation with *X. albilineans*

**Fig. 1:**
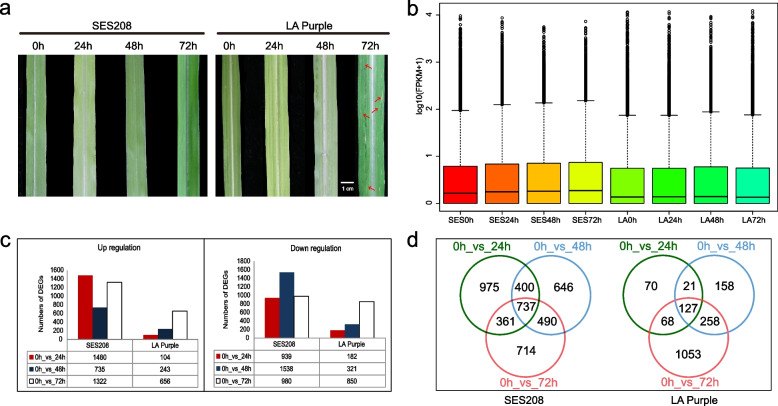
**a** Leaf scald symptoms observed in sugarcane varieties SES208 and LA Purple after inoculation with *Xanthomonas albilineans*. Pencil line streak (red arrow). **b** Box diagram of gene expression (log10(FPKM+ 1)). **c** The numbers of differentially expressed genes (DEGs) with increased or decreased transcript abundance in SES208 and LA Purple (|FDR| > 2,*p*-value< 0.005). **d** Venn diagrams of DEGs in SES208 and LA Purple, respectively. SES: SES208; LA: LA Purple. 0 h: uninfected plants; 24 h, 48 h, and 72 h represent 24, 48, and 72 hours post inoculation of sugarcane with *X. albilineans*, respectively

To investigate the differences in resistance to leaf scald caused by *X. albilineans* between the sugarcane cultivars SES208 (*S. spontaneum*), a disease-resistant variety, and LA Purple (*S. officinarum*), a disease-susceptible variety, we experimentally inoculated plants with *X. albilineans.* We observed the phenotypic differences between inoculated and uninoculated plants of these two species and collected leaf tissue samples at 24 hpi (hpi: hours post inoculation), 48 hpi, and 72 hpi for subsequent RNA extraction. Plants of SES208 and LA Purple exhibited different responses to inoculation with *X. albilineans.* Although there were no leaf scald symptoms on SES208 plants after infection, there were thin streaks on the leaves. LA Purple plants exhibited symptoms of leaf scald at 72 hpi infection (Fig. 1a), thus confirming the differences between LA Purple and SES208 in resistance to leaf scald caused by *X. albilineans*.

### Identification of DEGs and core conserved DEGs in resistant and susceptible sugarcane cultivars in response to inoculation with *X. albilineans*

Global gene expression profiles in leaves of SES208 (*S. spontaneum*) and LA Purple (*S. officinarum*) inoculated with *X. albilineans* and uninoculated control plants were analyzed at 24 hpi, 48 hpi, and 72 hpi. In general, global gene expression in SES208 was higher than that in LA Purple (Fig. 1b). Transcriptome comparisons at each time point were made within each of the cultivars. Compared to time point 0, at 24 hpi, transcripts of 1480 DEGs were more abundant and 939 DEGs were less abundant in leaves of SES208 while transcripts of 104 DEGs were more abundant and 182 DEGs were less abundant in leaves of LA-Purple (Fig. 1c). Trends in the number of genes with increased transcript abundance and those with decreased transcript abundance were similar between 0 and 72 hpi in LA Purple but differed during this time period in SES208 (Fig. 1c). Between 0 and 48 hpi, SES208 showed 735 genes with increased transcript abundance and 1538 genes with decreased transcript abundance (Fig. 1c). The number of genes with increased transcript abundance in SES208 was significantly greater than that in LA Purple (Fig. 1c). The highest number of genes with increased transcript abundance in SES208 occurred at 24 hpi, but in LA Purple occurred at 72 hpi (Fig. 1c). The highest number of DEGs with decreased transcript abundance occurred at 48 hpi in leaves of SES208 and at 72 hpi in leaves of LA Purple (Fig. 1c). A total of 4323 DEGs were expressed in leaves of SES208 in response to *X. albilineans*, nearly twice the number of DEGs in LA Purple in response to this pathogen (Fig. 1d). The expression of 975, 646, and 714 DEGs specifically changed at 24, 48, and 72 hpi, respectively, in SES208, while the expression of 70, 158, and 1053 DEGs specifically changed in LA Purple at 24, 48, and 72 hpi, respectively (Fig. 1d). These results indicate that the transcript expression of more genes in SES208 than in LA Purple changed rapidly during infection with *X. albilineans*.

**Fig. 2:**
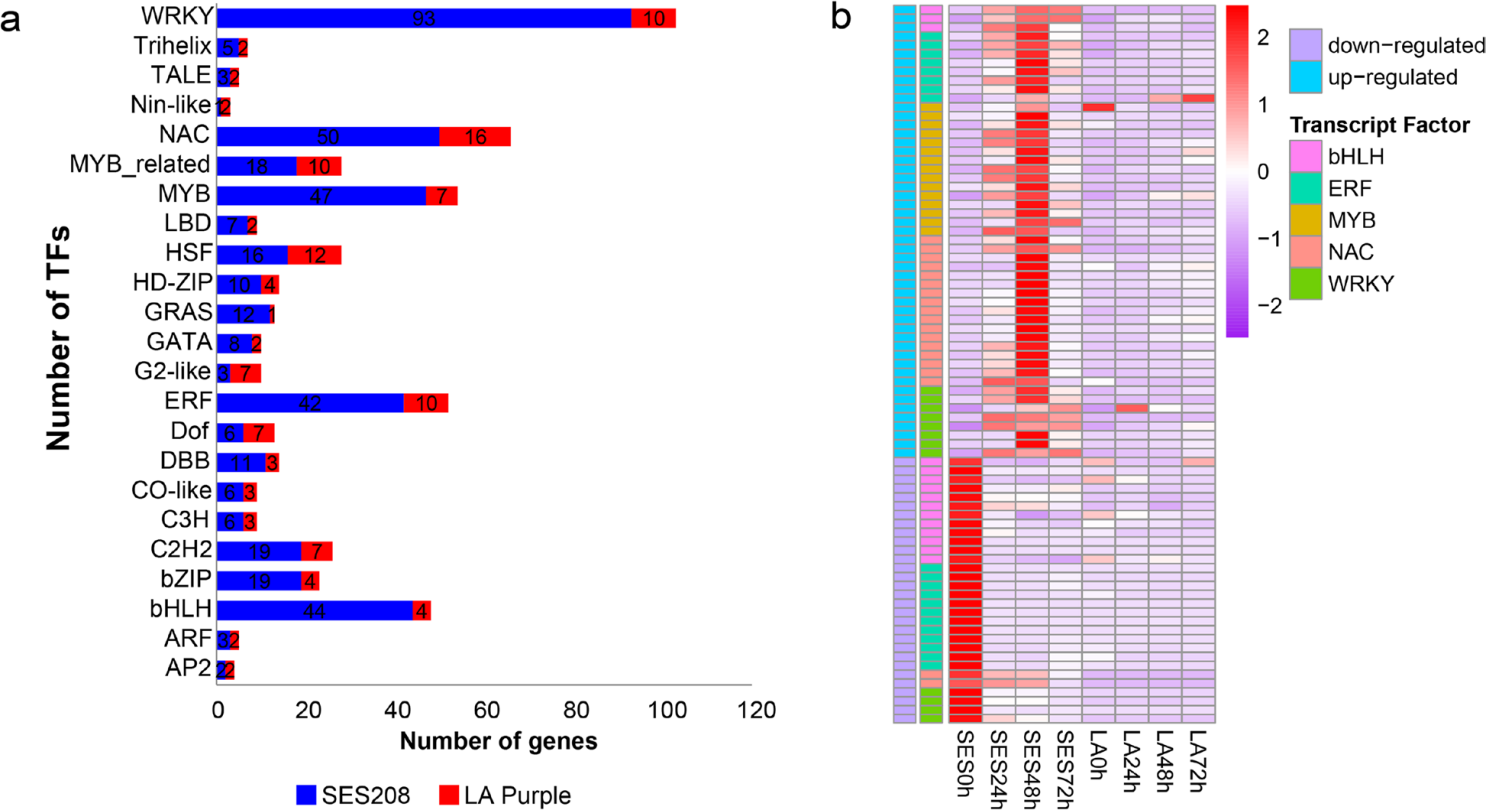
Transcription factors (TFs) encoded by DEGs in sugarcane varieties SES208 and LA Purple after inoculation with *Xanthomonas albilineans*. **a** The number of TFs encoded by DEGs. **b** Heat map of TF expression after inoculation with *X. albilineans*. Expression values were calculated as the relative expression of genes (log2 Fold Change from − 2 to + 2). SES: SES208; LA: LA Purple. 0 h: uninfected plants; 24 h, 48 h, and 72 h represent 24, 48, and 72 hours post inoculation of sugarcane with *X. albilineans*, respectively

Further, we annotated the genes encoding TFs among the DEGs responding to inoculation with *X. albilineans* in LA Purple and SES208. We identified genes representing a total of 23 TF families in both cultivars, and found that more TF families exhibited changed transcript expression in SES208 than in LA Purple (Fig. 2a). It is worth noting that gene families encoding WRKY, NAC, MYB, ERF, and bHLH were significantly overrepresented among DEGs in SES208 inoculated with *X. albilineans*. All DEGs encoding TFs showed higher expression in SES208 than in LA Purple except for three genes encoding ERF, MYB, and WRKY, respectively (Fig. 2b). The transcript abundances of 15 genes encoding MYBs, 17 encoding NACs, 8 encoding ERFs, and 8 encoding WRKYs significantly increased, while the transcript abundances of 12 genes encoding bHLHs and 12 encoding ERFs significantly decreased in SES208 compared to LA Purple after inoculation with *X. albilineans* (Fig. 2b), suggesting that members of these TF families might be involved in the regulation of responses to this pathogen in sugarcane.

### GO term and KEGG pathway enrichment analysis of DEGs responding to inoculation with *X. albilineans* in SES208 and LA purple

**Fig. 3:**
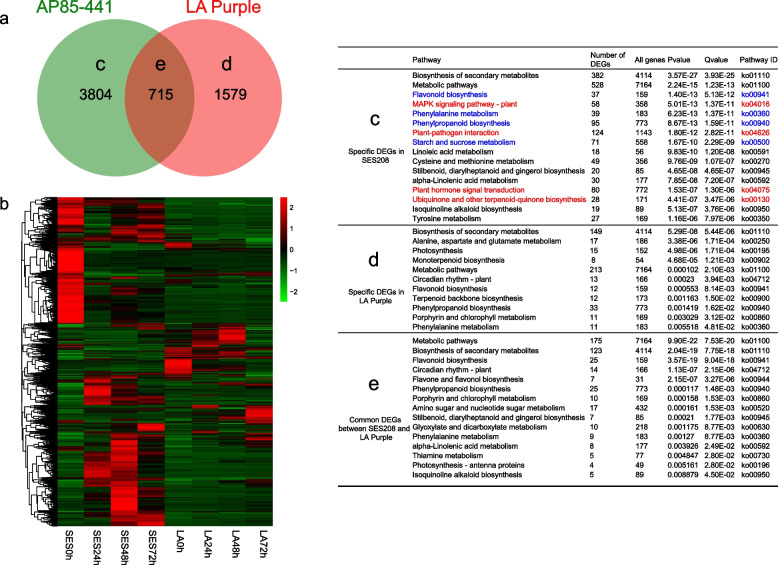
Analysis of differential gene expression in sugarcane varieties SES208 and LA Purple after inoculation with *Xanthomonas albilineans*. **a** Venn diagrams of DEGs between SES208 with LA Purple. **b** Heat map of conserved core DEGs expressed in common between SES208 with LA Purple after inoculation with *Xanthomonas albilineans*. **c-e** Numbers of DEGs in major KEGG pathways after inoculation with *Xanthomonas albilineans*. **c** Specific DEGs in SES208. The red font represents the pathway specifically enriched in SES208; The blue font indicates that the number of DEGs in the enriched pathway in SES208 is greater than that in LA Purple. **d** Specific DEGs in LA Purple. **e** DEGs expressed in common between SES208 and LA Purple. Expression values were calculated as relative expression of genes (log2 Fold Change from − 2 to + 2). SES: SES208; LA: LA Purple. 0 h: uninfected plants; 24 h, 48 h, and 72 h represent 24, 48, and 72 hours post inoculation of sugarcane with *X. albilineans*, respectively

To further analyze the possible functions of DEGs during *X. albilineans* infection in LA Purple and SES208 sugarcane, KEGG and GO analyses were performed. A total of 6098 DEGs in SES208 and LA Purple were significantly enriched in 119 KEGG pathways (Fig. 3a). By cross-comparison of all DEGs between SES208 and LA Purple, we found that these two cultivars shared 715 DEGs across the comparison, while 3804 showed specifically changed expression in SES208 and 1579 DEGs showed specifically changed expression in LA Purple (Fig. 3a). We also found that the overall expression of DEGs responding to *X. albilineans* was higher in SES208 than in LA Purple (Fig. 3b). KEGG analysis was performed on the core DEGs and cultivar-specific DEGs in SES208 and LA Purple. In SES208, we found that 3804 cultivar-specific DEGs were enriched in 110 pathways, particularly in plant-pathogen interaction (Fig. 3c). Notably, 58, 124, 80, and 28 genes were significantly enriched for pathways including MAPK signaling, plant-pathogen interactions, plant hormone-mediated signal transduction, and the biosynthesis of ubiquinone and other terpenoid quinones, respectively. Another 95, 71, 39, and 37 DEGs were mainly concentrated in functions such as the biosynthesis of phenylpropanoids, metabolism of starch and sucrose, metabolism of phenylalanine, and the biosynthesis of flavonoids, respectively (Fig. 3c). In LA Purple, 1579 cultivar-specific DEGs were also significantly enriched for terms in metabolic pathways and the biosynthesis of secondary metabolites (Fig. 3d). A total of 715 DEGs in common between SES208 and LA Purple were enriched in 76 pathways and 298 DEGs were enriched for terms related to metabolic pathways and secondary metabolite biosynthesis (Fig. 3e). Our GO term enrichment analysis showed that, compared to LA Purple, DEGs in SES208 were significantly enriched for terms in the biological processes domain including those related to responses to stresses or plant hormones (Additional file 1).

### WGCNA analysis of coexpressed gene modules responding to inoculation with *X. albilineans* in SES208 and LA purple

**Fig. 4:**
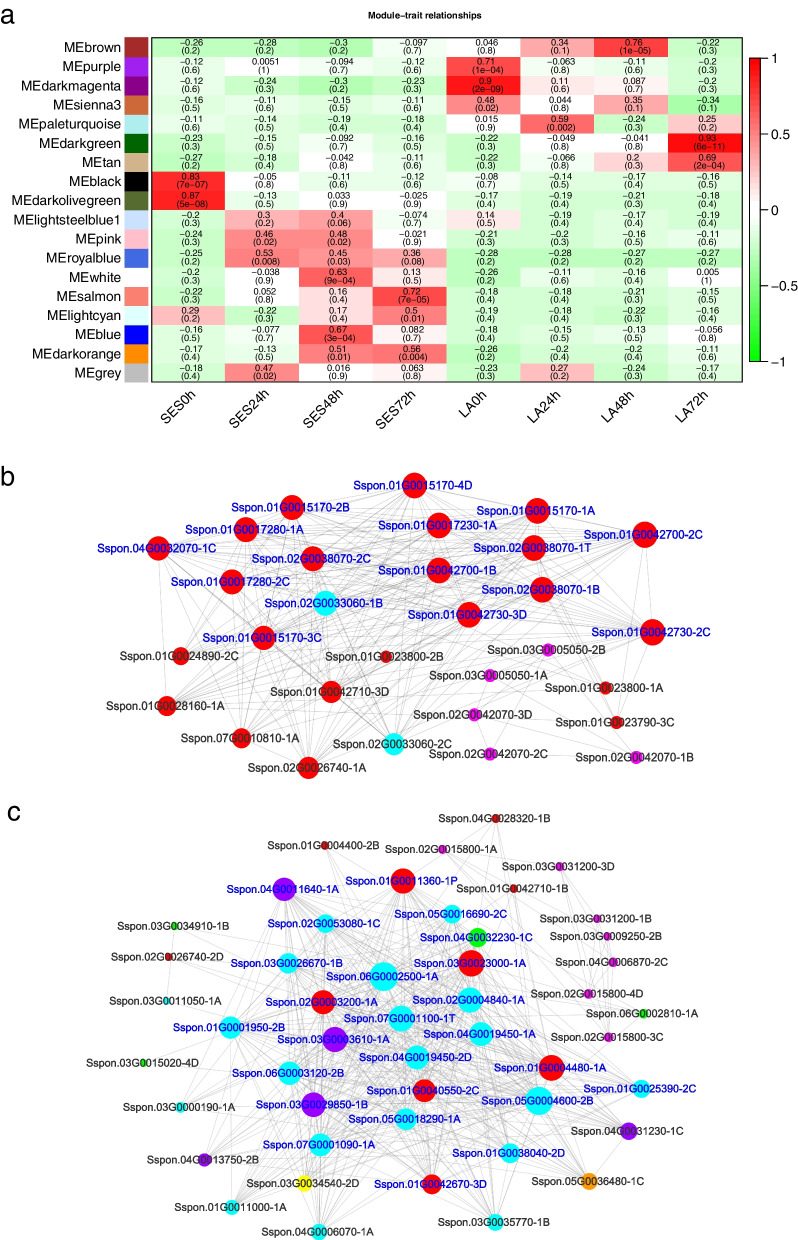
Weighted gene co-expression network analysis (WGCNA) of sugarcane varieties SES208 and LA Purple after inoculation with *Xanthomonas albilineans*. **a** Heat map of the correlation between WGCNA modules in SES208 and LA Purple. SES: SES208; LA: LA Purple. The left lane indicates 18 modules. The right lane indicates the module-trait correlation from − 1 to 1. 0 h: uninfected plants; 24 h, 48 h, and 72 h represent 24, 48, and 72 hours post inoculation of sugarcane with *X. albilineans*, respectively. **b** The co-expression networks in SES208 in MEblack module. **c** The co-expression networks in SES208 in MEdarkolivegreen module. Light blue: plant pathogen interaction; Purple: MAPK signaling pathway; Red: plant hormone signal transduction; Orange: terpenoid quinone biosynthesis; Green: phenylpropanoid biosynthesis; Pink:starch and sucrose metabolism. The blue font indicates hub genes

To identify the key regulatory genes related to *X. albilineans* resistance in SES208 and LA Purple, a WGCNA was performed. A total of 6098 DEGs in SES208 and LA Purple were chosen for constructing the network, and of the 18 modules with correlated expression detected by WGCNA, seven had correlation coefficients higher than 0.7 (*p* < 0.001) (Fig. 4a). DEGs in SES208 enriched in the MEblack module (*r* = 0.83) and the MEdarkolivegreen module (*r* = 0.87) were involved in plant hormone signaling, MAPK signaling, plant-pathogen interactions, biosynthesis of terpenoid quinones, metabolism of starch and sucrose, and phenylpropanoid biosynthesis (Additional file 2). These genes were then chosen to construct coexpression networks to identify candidate regulatory genes that respond to *X. albilineans* in SES208. Our co-expression network indicated 16 and 25 hub genes in the MEblack module and the MEdarkolivegreen module, respectively (Fig. 4b, c). These hub genes are enriched for putative functions in plant-pathogen interactions, MAPK signaling, and plant hormone signal transduction, all of which might be involved in regulating the responses of sugarcane to infection with *X. albilineans* (Fig. 4b, c).

### Allelic analysis of DEGs that respond to inoculation with *X. albilineans* in SES208 and LA purple

**Fig. 5:**
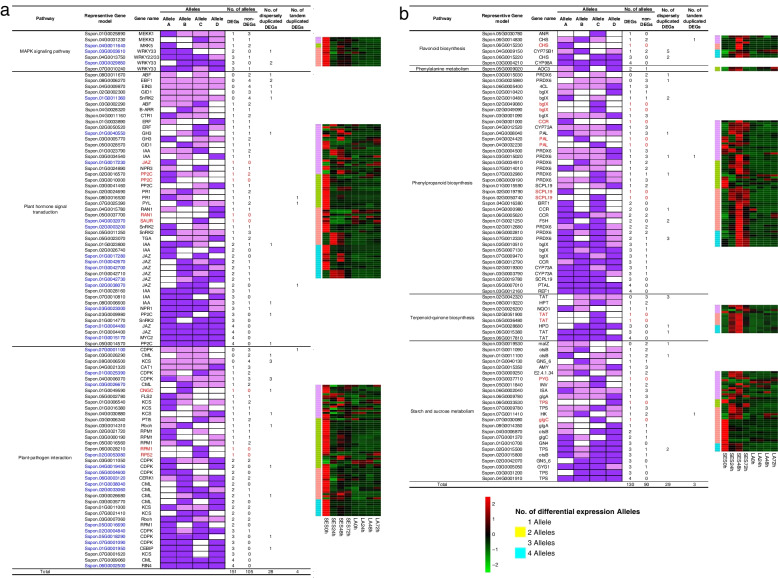
Alleles analysis of DEGs involved in major pathways with function annotation information and expression pattern between sugarcane varieties SES208 and LA Purple after inoculation with *Xanthomonas albilineans*. **a** Alleles analysis of DEGs involved in MAPK signaling pathway, plant-pathogen interaction and plant hormone signal transduction. **b** Alleles analysis of DEGs in phenylpropanoid biosynthesis, phenylalanine metabolism, flavonoid biosynthesis and terpenoid quinone biosynthesis. The blue font indicates hub genes in Fig. 4. Genes shown in red font exhibited expression of one specific allele. The expression value was calculated by relative expression of genes (log2 Fold Change from − 2 to + 2). SES: SES208; LA: LA Purple. 0 h: uninfected plants; 24 h, 48 h, and 72 h represent 24, 48, and 72 hours post inoculation of sugarcane with *X. albilineans*, respectively

Allelic diversity including ASE (allele-specific expression) may be assessed in diploid or polyploid plants. ASE can lead to dramatic changes in gene expression profiles [32]. To identify ASE of defense response genes in sugarcane, we analyzed 345 DEGs from SES208 and LA Purple sugarcane that were identified in response to *X. albilineans* by KEGG pathway enrichment analysis (Fig. 5). According to the allele table https://www.life.illinois.edu/ming/downloads/Spontaneum_genome/Saccharum%20spont%20alleleTable-Jan%202019.csv [4], all of these 345 DEGs have one to four alleles and include dispersed duplicated genes and tandem duplicated genes. These 345 DEGs include a total of 281 differentially expressed alleles, 57 dispersed duplicated genes, and 7 tandem duplicated genes (Fig. 5). Many of the DEGs that might be involved in plant-pathogen interactions, MAPK signaling, and plant hormone signal transduction exhibited decreased transcript abundance after *X. albilineans* infection, indicating that the expression of these DEGs was likely negatively regulated in response to pathogen invasion (Fig. 5a). However, the transcript abundances of most DEGs in metabolic pathways increased, indicating that the expression of these DEGs was likely positively regulated in response to pathogen invasion (Fig. 5b).

Of these 345 DEGs, 21 exhibited expression of one specific allele (Fig. 5). We also examined the expression of differentially expressed alleles of DEGs with two, three, or four alleles in response to *X. albilineans*, and found that 64.86, 49.02, and 30.61% of the haplotypes were differentially expressed among these multi-allelic DEGs, respectively, indicating an allelic dominance effect in these two cultivars responding to *X. albilineans* treatment (Fig. 5; Additional file 3). After analyzing their transcript expression in SES208, we found that 85 out of 281 DEGs exhibited ASE, 42 out of these 85 DEGs expressed alleles with increased transcript abundance, and 43 out of these 85 DEGs exhibited decreased transcript abundance for one differentially expressed allele at 24 hpi (Additional file 4). It is noteworthy that we identified 62 genes with two differentially expressed alleles, 93 genes with three differentially expressed alleles, and 44 genes with four differentially expressed alleles (Addition file 3). The transcript abundances of alleles of about 37.2% of the genes with two to four alleles were strictly up-regulated at 24 hpi in response to *X.albilineans* in SES208 (Additional file 4)*.* Thus, our results indicate that ASE is associated with resistance to infection by *X. albilineans* in SES208. Further, we discovered that the genes exhibiting ASE showed similar expression patterns in response to *X. albilineans* in SES208 (Additional file 4). Further, 19 DEGs showed increased transcript abundances and 38 DEGs showed decreased transcript abundances at 24 hpi in dispersed duplicated DEGs (Additional file 5). The transcript abundances of five tandem duplicated DEGs showed decreased transcript abundances at 24 hpi and two tandem duplicated DEG showed increased transcript abundances at 48 hpi in SES208 during *X. albilineans* infection (Additional file 5). These results indicated the expression of these alleles, dispersed duplicated genes, and tandem duplicated genes showed significant and consistent responses to infection with *X. albilineans*.

### Potential functions of major metabolic pathways to *X. albilineans* infection in sugarcane

Our KEGG analysis of DEGs responding to inoculation with *X. albilineans* infection identified a total of 165 DEGs between SES208 and LA Purple that might participate in the biosynthesis of terpenoid quinones, phenylpropanoids, and flavonoids, or the metabolism of starch and sucrose or phenylalanine, among which 122 of 165 DEGs exhibited high transcript abundances and 43 out of 165 DEGs exhibited low transcript abundances in SES208 compared to LA Purple at 24 hpi in response to *X. albilineans* infection (Fig. 5b).

The transcript abundances of DEGs encoding the key enzymes involved in flavonoid biosynthesis, phenylalanine metabolism, and terpenoid quinone biosynthesis were mainly increased in SES208 compared to LA Purple in response to *X. albilineans* infection and the alleles of these DEGs showed the same expression patterns (Fig. 5b). Of the DEGs putatively involved in the phenylpropanoid biosynthesis pathway, the transcript abundances of 81% were increased at 24 hpi or 48 hpi in SES208 but were less highly expressed in LA Purple (Fig. 5b). The transcripts of all alleles of the beta-glucosidase(*BglX*) and Bright Trichomes 1 (*BRT1*) genes, which are both essential in phenylpropanoid synthesis, increased significantly after infection with *X. albilineans* in SES208 but not in LA Purple (Fig. 5B; Additional file 6). The CYP450 family is involved in the synthesis and metabolism of phenylpropanoids [33] and one of its members, *CYP73*, also showed increased transcript expression after 24 hpi in SES208 (Fig. 5B; Additional file 7). The *CYP75* gene family plays an important role in flavonoid biosynthesis in plants [34]. Members of the CYP 98 family catalyze the 3-hydroxylation step in the phenylpropanoid pathway (add reference). Interestingly, all alleles of these two genes showed increased transcript abundance after 24 hpi in SES208 (Fig. 5B), indicating that their expression responds positively to infection with *X. albilineans* in SES208. However, the expression of these two genes showed little change in LA Purple (Fig. 5B), which could be consistent with the stronger resistance of SES208 than LA Purple to infection with *X.albilineans*.

DEGs associated with starch and sucrose metabolism pathways showed differential expression trends in SES208 and LA Purple after *X. albilineans* infection. For example, *glucan endo-1,3-beta-glucosidase 4* (*GN4*) and *glucose-1-phosphate adenylyltransferase* (*glgC*) exhibited increased transcript expression in SES208 at 24 hpi (Additional file 7). However, glycogenin (*GYG1*) exhibited decreased transcript expression at 24 hpi (Additional file 6). The transcript abundances of *otsB*, which encodes trehalose 6-phosphate phosphatase, sharply decreased for five of eight alleles at 24 hpi in response to *X. albilineans* infection. The dynamic changes in the expression of the above DEGs in the starch and sucrose metabolism pathway might provide evidence of a direct relationship between higher *X. albilineans* resistance and lower biomass in SES208.

### Potential functions of the MAPK signaling pathway and plant-pathogen interaction pathway during *X. albilineans* infection

**Fig. 6:**
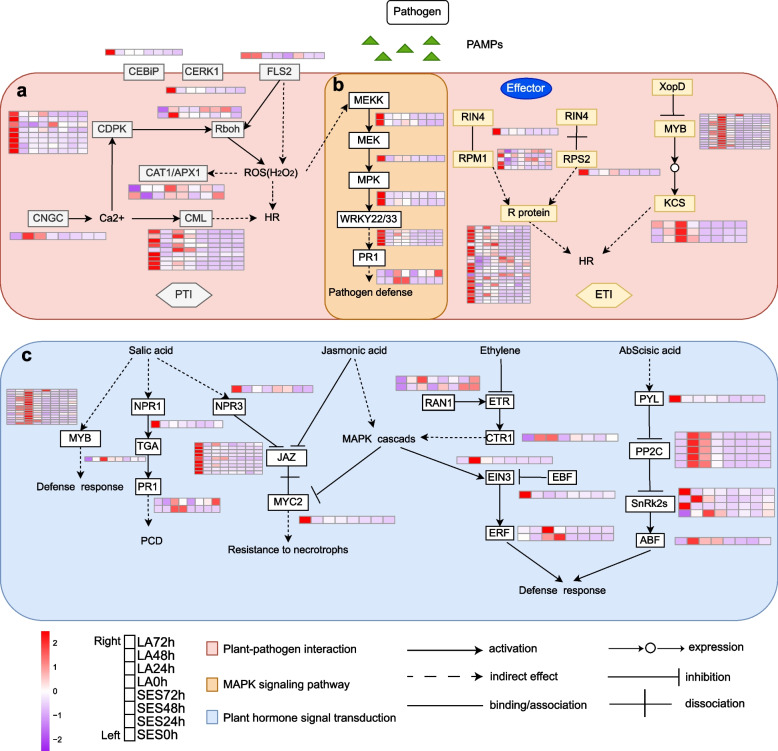
Models for regulation of gene expression in plant-pathogen interaction, MAPK signaling pathway, and plant hormone signal transduction in sugarcane varieties SES208 and LA Purple after inoculation with *Xanthomonas albilineans*. **a** Plant-pathogen interaction. **b** MAPK signaling pathway. **c** Plant hormone signal transduction. Expression values were calculated by relative expression of genes (log2 Fold Change from − 2 to + 2). SES: SES208; LA: LA Purple. 0 h: uninfected plants; 24 h, 48 h, and 72 h represent 24, 48, and 72 hours post inoculation of sugarcane with *X. albilineans*, respectively

Based on the above results, we identified candidate genes from the plant-pathogen interaction and MAPK signaling pathways whose expression responds to *X. albilineans* infection. In PTI, respiratory burst oxidase homologues (RBOHs) are a major source of reactive oxygen species (ROS) during plant-microbe interactions [35, 36]. We found that the *Rboh* gene exhibited significantly increased expression in response to inoculation with *X. albilineans* in SES208 (Fig. 6a). Further, transcript expression of the catalase1/ascorbate peroxidase1 (*CAT1*/*APX1*) genes related to cell death and H_2_O_2_ production increased, which might induce MAPK signaling to protect plant cells from oxidative stress and death. In the early stages of *X. albilineans* infection of sugarcane, the transcript expression of *SsMEKK*, *SsMKK*, and *SsMPK* decreased (Fig. 6b). The transcript expression of *SsWRKY22* and *SsWRKY33,* TF-encoding core genes with two and three alleles, respectively, decreased sharply in response to infection with *X. albilineans*, suggesting that the decreased expression of these genes could be an important aspect of the sugarcane response to *X. albilineans* (Fig. 5a; Fig. 6b)*.* The expression of genes encoding cyclic nucleotide gated channels (*CNGCs*) that are normally involved in the regulation of Ca^2+^ channels and stomata increased in SES208, while the expression of *CNGCs* showed no response in LA Purple (Fig. 6a). The expression of the majority of genes encoding *CDPK* and *CML,* except for *Sspon.03G0026290-3P* in SES208*,* dramatically decreased at 24 hpi (Fig. 6a).

The transcript abundance of the ethylene receptor ripening inhibitor (*RIN*) gene decreased in SES208 but not in LA Purple (Fig. 6a). The disease resistance genes *RPM1* (RESISTANCE TO *P. SYRINGAE* PV MACULICOLA 1) and *RPS2* (RIBOSOMAL PROTEIN S2), which exhibit ASE, decrease in transcript expression after *X. albilineans* infection in SES208. In contrast, downstream resistance genes involved in R gene-mediated plant disease resistance responses showed significantly decreased expression (Fig. 6a). Further, the 3-ketoacyl-CoA synthase (*KCS*) genes involved in the synthesis of ultra-long-chain fatty acids increased sharply at 24 hpi (Fig. 6a). These results indicate that differential expression of genes involved in the PTI and ETI signaling pathways is likely essential for *X. albilineans* response in SES208.

### Potential functions of plant hormone signal transduction in resistance to *X. albilineans* infection

The plant hormones SA, JA, ET, and ABA act in pathways that protect plants against infection with pathogens [37]. NPR1-LIKE PROTEIN 3 (NPR3) is an important regulator of plant SAR signaling, and we found that the transcript expression of the *NPR3* genes decreased sharply in SES208 and LA Purple at 24 hpi (Fig. 6c; Additional file 7). In SES208, the *transglutaminase* (*TGA*) gene downstream of the SA pathway exhibited significantly increased transcript expression that peaked at 48 hpi in SES208, but showed a slight decrease at 24 hpi in LA Purple (Fig. 6c; Additional file 7). The transcript abundance of the downstream gene *PR1* (*pathogenesis-related protein 1*), a core R gene with ASE, increased in both SES208 and LA Purple in response to *X. albilineans* infection (Fig. 6c). The *JAZ* (*jasmonate ZIM domain*) gene functions during the regulation of responses involving the plant hormone JA [38]. At 48 hpi and 72 hpi, the transcript abundances of *JAZ* genes were significantly increased in SES208 (Fig. 6c). All four alleles of *MYC2* showed decreased expression at 24 hpi in SES208 (Fig. 5a; Fig. 6c; Additional file 7).

In the ethylene signal transduction pathway, response to antagonist 1 (RAN1) helps ethylene receptor (ETR) to interact with ethylene [39]. After *X. albilineans* infection, the expression of *ETR* decreased in LA Purple, but increased in SES208 (Fig. 6c). Transcript expression of the *EBF1* (*EIN3-BINDING F BOX PROTEIN 1*) gene decreased significantly in SES208, but there was no obvious change in the transcript abundance of this gene in LA Purple (Fig. 6c; Fig.8). The expression of *ETHYLENE-INSENSITIVE3* (*EIN3*) was likely inhibited by *EBF1* in SES208 while the expression of *ETHYLENE RESPONSE FACTOR* (*ERF*) genes increased in both SES208 and LA Purple (Fig. 6c). Transcripts encoding PYL (pyr1-like), an ABA receptor that is involved in the sensing and transduction of ABA signals [40] were expressed at lower levels after infection in SES208. However, genes encoding type 2C protein phosphatases (PP2C) were all expressed at higher levels at 24 hpi, and the downstream genes encoding SnRK2 (class III SNF-1-related protein kinase 2) exhibited increased expression of two genes and decreased expression of two other genes after inoculation of SES208 with *X. albilineans* (Additional file 7). Further, the expression of genes encoding abscisic acid-responsive TFs (ABF) increased and their transcripts exhibited increased abundance at 24 hpi in SES208 (Fig. 6c).

## Discussion

### Analysis of sugarcane cultivars transcriptomes during *X. albilineans* infection

We constructed 24 cDNA libraries to elucidate genetic responses that are triggered by *X. albilineans* infection in the sugarcane species *S. spontaneum* and *S. officinarum*. Comparative transcriptome and functional enrichment analyses of SES208 and LA Purple revealed sharp *X. albilineans* infection-induced gene expression changes in SES208 compared to LA Purple. These changes involved genes in key plant defense pathways such as plant-pathogen interaction, MAPK signaling, and plant hormone function pathways in addition to metabolic pathways for flavonoid, phenylpropanoid, and terpenoid quinone biosynthesis as well as for phenylalanine, starch, and sucrose metabolism.

Polyploidy results in changes in cellular structure, function [41, 42], and allelic diversity [43] that result in dramatic changes in gene expression profiles [44, 45], such as ASE [32]. Here, we identified a total of 345 DEGs involved in *X. albilineans* resistance in SES208 (Fig. 5). The hub DEGs identified were candidates that might be crucial for responses to *X. albilineans* infection in sugarcane. Among these 345 DEGs, 85 exhibited ASE while 196 showed balanced allelic expression (Additional file 4). We suggest that ASE in SES208 is largely a genotype-specific phenomenon and might be common in some individual wild species. Notably, the expression patterns of genes with multiple alleles were similar, which indicates that these alleles might act synergistically in resistance to *X. albilineans*. We predict that the genes showing ASE in SES208 reflect the breeding history of sugarcane, and might have undergone greater selection pressure. Furthermore, the occurrence of ASE among these defense genes might be associated with traits inherited from wild genotypes as discussed previously [46]. The higher frequency of ASE in defense-responsive genes is consistent with observations in wheat, in which many such genes exhibit homologous gene expression bias in plants attacked by pathogenic fungi [47]. Sugarcane hybridization and breeding have long focused mainly on selection for disease resistance and cultivars thus vary widely in their response to pathogen infection.

Transcriptional regulation is an important aspect of gene expression regulation in plants that controls many crucial biological processes via gene regulatory networks [48, 49]. Transcripts encoding five types of TFs including WRKY, NAC, MYB, ERF, and bHLH were enriched in SES208 in response to *X. albilineans* infection, which suggests that these TFs could be directly or indirectly involved in resistance to *X. albilineans*. Previous studies have indicated that WRKY TFs can interact with receptors for PTI and become involved in MAPK signaling to activate or regulate gene expression [50]. The decreased transcript expression of *SsWRKY*s in SES208 indicates that these genes might be involved in some regulatory responses during defense to *X. albilineans* (Fig. 2; Fig.8). NAC TFs *ANAC019* and *ANAC055* might be involved in plant defense responses mediated by JA and in regulation of the expression of the JA-induced defense genes *VSP1* and *LOX2* [51]. R2R3-MYB TFs such as *AtMYB96* [50] and *OsMYB4* [52] could increase *P. syringae* resistance and increase the expression of PR protein-encoding genes in *Arabidopsis* and rice, respectively. The transcript expression of about 90% of the *SsNAC*s and all of the *SsMYB*s in SES208 increased, suggesting that these TFs might activate gene expression during resistance to *X. albilineans* (Fig. 2). ERF19 plays a role with NINJA in ERF19-mediated regulation of *Arabidopsis* innate immunity [53]. *SsERF* genes show ASE and all increase in transcript abundance in SES208, suggesting that they might activate gene expression to enhance resistance to *X. albilineans* (Fig. 2). The bHLH transcription factor *GmPIB1* increases soybean resistance to *P. sojae* infection by inhibiting the expression of *GmSPOD1* [54]. Further, the bHLH TF HBI1 negatively regulates some aspects of disease defense responses in *Arabidopsis* [55]. However, the transcript abundances of about 85% of *SsbHLH*s actually decreased in SES208, suggesting that the expression of the bHLH TFs themselves might be regulated negatively during responses to *X. albilineans* in sugarcane. This might also indicate that increased or decreased transcript expression of genes encoding different classes of TFs affect various resistance responses to *X. albilineans* in SES208 (Fig. 2).

### Plant-pathogen interaction pathway responsive *X. albilineans* infection in sugarcane

**Fig. 7:**
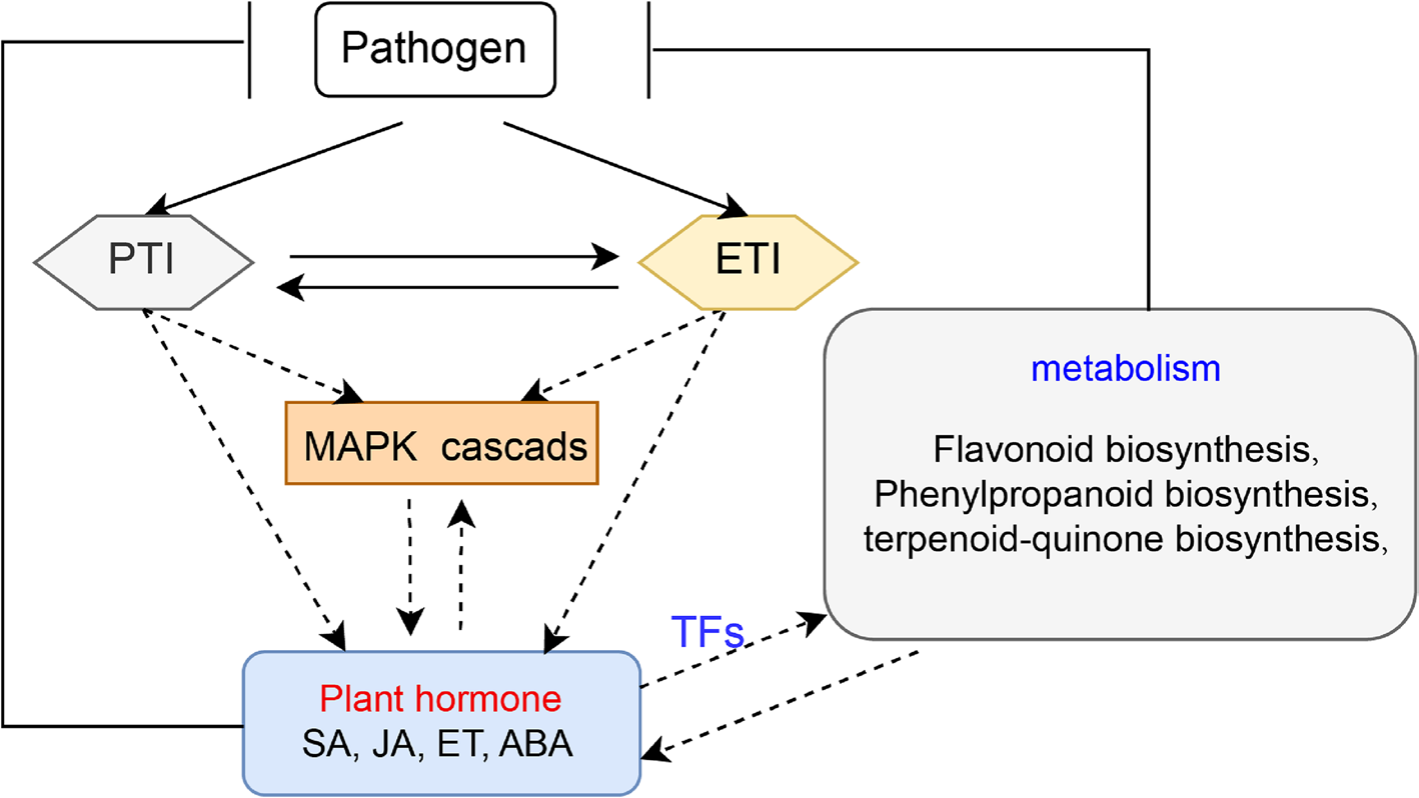
Hypothetical model of the molecular mechanism of the response to *X. albilineans* in sugarcane

**Fig. 8:**
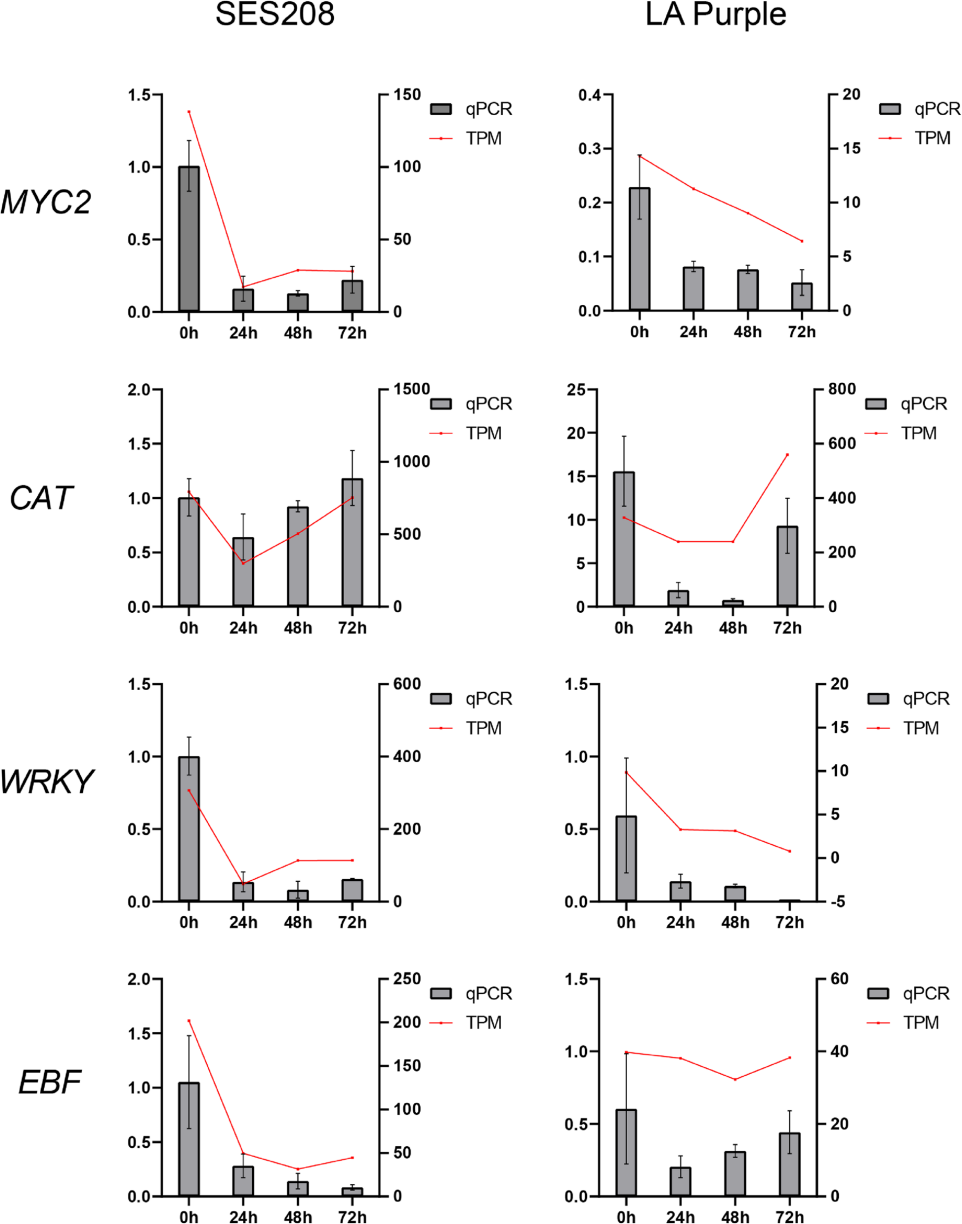
Verification by qRT-PCR of the expression patterns of four DEGs involved in different pathways during infection of sugarcane with *Xanthomonas albilineans*. The right *y*-axis indicates FPKM value; the left *y*-axis indicates relative expression; the *x*-axis indicates different time periods after infection. 0 h: uninfected plants; 24 h, 48 h, and 72 h represent 24, 48, and 72 hours post inoculation of sugarcane with *X. albilineans*, respectively

Plant immunity requires the action of multiple receptors and ligands to provide adequate protection against pathogen attack. In the early stage of oxidative burst when plants are infected by pathogenic microorganisms, they sense PAMPs, abiotic stress, and extracellular signals through surface-localized PRRs [56]. Flagellin receptor *FLS2* can recognize bacterial proteins [57] and elevate ROS production [58]. APX1 is a key factor in removing ROS produced in response to stresses and disease progression in plants [59]. In addition, CAT can also decompose H_2_O_2_ to generate O_2_, and trigger benzoic acid synthesis for generation of SA, leading to the elaboration of a systemic defense response (Fig. 7). CAT activity in transgenic tobacco can result in serious HR and effectively control bacterial infection [60, 61]. The expression of the *SsCAT1* gene decreased continuously at 24 hpi upon *X. albilineans* infection (Fig. 8), and the transcript abundance of two *SsAPX1* genes increased at 24 hpi in SES208 (Fig. 6a). These results demonstrate that Ss*WRKY* genes might negatively regulate the expression of *SsPR1* and plants might mainly rely on *SsAPX1* to generate H_2_O_2_ in early stages of infection and then rely on *SsCAT1* activated by the MAPK signaling pathway during later stages. CEBiP and CERK act as LysM-RP and LysM-RK proteins, respectively, and are also regarded as PRRs [62]. Interestingly, the response of CEBiP to the pathogen in SES208 and LA Purple was similar to that of OsLYP4 and OsLYP6 in response to *X. oryzae pv. oryzae* (PGN_Xoo_), *X. oryzae pv. oryzaecola* (PGN_Xoc_), and *Pseudomonas syringae pv. tomato (Pto)* DC3000 (PGN_Pto_) in rice. OsLYP4 and OsLYP6 are normally complexed but can dissociate and when complexed with OsCERK can sense bacterial peptidoglycan (PGN). The susceptibility of transgenic plants was positively correlated with the transcript abundances of *OsLYP4* and *OsLYP6* genes [63]. At 24 hpi, the transcript abundance of *CEBiP* is lower in SES208 and higher in LA Purple (Additional file 7).

As a cation channel protein, CNGC can participate in the regulation of Ca^2+^ influx to prevent pathogen invasion [64]. In SES208, the expression of *CNGS* transcripts showed an increasing trend. If CNGS participates in *X. albilineans* resistance in SES208, it might act to open calcium channels, promote Ca^2+^ influx, and activate intracellular disease resistance genes (Fig. 6a; Additional file 7). Calcium-dependent protein kinases (CDPKs) can be activated by Ca^2+^ [65] and overexpression of the *CPK4* gene in tobacco could enhance the transcription of SAR-related genes, lead to HR, and improve the broad-spectrum disease resistance of plants [66]. In addition, the increase in calcium can directly or through *CaM/CML*, result in the phosphorylation or dephosphorylation of TFs [67]. In the early stages of infection in sugarcane, metabolites from pathogenic bacteria might inhibit the binding of SsCML and SsCDPK to calcium ions, resulting in decreased expression of *SsCML* and *SsCDPK* transcripts (Additional file 7). Expression of *SsRboh* transcripts increases, which might enhance production of reactive oxygen species (H_2_O_2_) (Fig. 6a). The processes above constitute our proposed model for a pathogen-associated molecular PTI response that might occur during the response of SES208 against pathogens.

When a pathogen suppresses or acts on PTI by secreting effector proteins into the host, R proteins can sense or recognize the effector protein, thereby triggering ETI [15]. During the ETI response, effectors (*Avirulence*, *Avr*) activate the expression of numerous genes encoding disease-resistance proteins that specifically recognize the effector and allow a resistance response [15]. The RPM1 protein can recognize the RIN4 protein that becomes phosphorylated due to the presence of the effectors AvrRPM1 and AvrB, which in turn results in the HR [68]. The *RPS2* gene that functions in disease resistance in *Arabidopsis* was used to identify a virulence gene *avrRpt2* and to select the mutants with altered resistance to *Pseudomonas syringae*, a bacterial plant pathogen [69]. In SES208, the transcript abundances of *SsRIN4*, *SsRPM1*, *SsRPS2*, and genes encoding R proteins significantly decreased within 0-24 hpi (Fig. 6a). It is possible that when plants are strongly attacked by pathogenic organisms, these genes cannot be expressed quickly enough to resist invasion by pathogens. The expression of these genes responds relatively weakly to pathogen invasion and a slight HR occurs. The protein encoded by *XopD* from *X. campestris pv*. *campestris* strain B100 targets the MYB30 TF in *Arabidopsis* that activates plant defense and cell death induced by bacteria [70]. In our analysis of TFs, we observed increased transcript expression for 15 genes encoding MYB TFs (Fig. 2; Fig. 6a). Transcript abundances of *MYB* genes and *KSC* both increased in SES208 (Fig. 6a), which suggest that MYBs might act as positive regulators of *KCS* to activate SES208 defenses against *X. albilineans* infection.

### Possible roles of plant hormone-mediated signal transduction genes responsive to *X. albilineans* infection in sugarcane

Both PTI and ETI can cause increased biosynthesis of SA and defense phytohormones that mediate SAR (Fig. 7) [71]. The SA receptor NPR3 is known to promote the degradation of the transcriptional repressor JAZ, thereby activating JA synthesis and inducing JA-responsive gene expression to allow plants to resist pathogens [72]. In SES208, the expression of *Sspon.01G0024890-2C* (*SsNPR3*) decreased, which suggests that defense responses in SES208 might not depend on this signaling pathway (Fig. 6c; Additional file 7). *MYC2* is a positive regulator in the JA signal pathway [73]. At 0 hpi, the abundances of *MYC2* and *JAZ* transcripts were significantly higher in SES208 than in LA Purple (Fig. 8), but the expression of both these genes decreased in response to *X. albilineans* in SES208 (Fig. 6c; Additional file 7), suggesting that JAZ might interact with MYC to inhibit the transcription of JA-responsive genes. During infection of plants with viruses or other pathogens, HR responses are triggered by Avr/R protein interactions that can result in changes in the levels of disease defense-related hormones including ET, JA, NO, SA, and in the accumulation of ROS via MAPK signaling (Fig. 7) [74].

Some studies have shown that ABA can also have positive or negative regulatory effects during plant immunity [75, 76]. In our study, we regarded the *SsPYL* and *SsPP2C* genes as putative negative and positive regulators of responses to *X. albilineans* infection (Fig. 6c; Additional file 7). Therefore, we hypothesize that several members of the MAPK family might be activated directly or indirectly by the ABA core signaling pathway during response to *X. albilineans* infection (Fig. 7) [77]. The above results suggest that ETI and ABA might play necessary roles in response to *X. albilineans* infection in SES208.

### Major metabolic pathways responsive to *X. albilineans* infection in sugarcane

Studies of different plant metabolites have shown the potential defensive activity of these compounds against plant pathogens [24]. The phenylpropanoid pathway is involved in the production of flavonoids, lignans, phytoalexins, phenolic acids, coumarins, and stilbene, which are directly and indirectly involved in plant development and disease responses [78]. Thus, altered levels of JA, SA, and secondary metabolites might influence HR and other disease resistance-related processes (Fig. 7) [79]. We found that the transcript expression of essential genes *BglX* and *BRT1* encoding important enzymes in the phenylpropanoid biosynthesis pathway increased in SES208 during *X. albilineans* infection, which might have contributed to the defense of plants against this pathogen (Additional file 7). As important phenylpropanoid compounds, flavonoids and their derivatives have been shown to act as antimicrobial and signaling molecules during plant defense responses [80]. Enhanced levels of flavonoids in cotton suppress plant invasion by the fungal pathogen *Verticillium dahliae* [81]. The expression of flavonoid biosynthesis-related enzymes such as chalcone synthase (CHS), 5-O-(4-coumaroyl)-D-quinate 3′-monooxygenase (CYP98A/C3’H), flavonoid 3′-monooxygenase (CYP75B1) encoded by *SsCHS*, *SsCYP98A*, and *SsCYP75B1*, respectively, increased in SES208 after *X. albilineans* infection (Fig. 5b; Additional file 7), suggesting that increased flavonoid synthesis might be part of the defense response against *X. albilineans* infection in this sugarcane cultivar.

In maize, terpenoids represent another class of metabolites with disease defense functions in plants. Therefore, the higher expression of important genes in the terpenoid biosynthetic pathway and the increased accumulation of terpenoids have both been correlated with disease resistance [82, 83]. The transcript expression of four genes encoding 4-hydroxyphenylpyruvate dioxygenase (HPD) increased in SES208 after *X. albilineans* infection (Fig. 5b; Additional file 7), suggesting that the synthesis of terpenoids might aid defense against *X. albilineans* infection in SES208. Interestingly, many plant hormones, including cytokinins, ABA, and gibberellins are derived from terpenoids (Fig. 7) [84, 85].

During various metabolic processes, TFs can regulate the transcript abundances of genes that encode the regulatory proteins and structural enzymes that participate in the biosynthesis of secondary metabolites. Six major TF families including AP2/ERF, bHLH, MYB, NAC, WRKY, and bZIP are involved in the metabolism of terpenoids in plants [86], and the MYB-bHLH-WD40 complex regulates flavonoids bio-synthesis in plants [87, 88](Fig.7). Overall, the expression of DEGs encoding TFs and enzymes involved in primary and secondary metabolism and hormone signaling was more rapidly and strongly induced in SES208 than in LA Purple after inoculation with *X. albilineans*. Increased expression of transcripts related to the accumulation of metabolites also significantly increased in SES208, which is consistent with some previous findings that altered metabolite abundances can be associated with greater disease resistance in plants [89, 90]. These results suggest avenues for future research related to the effects of metabolic changes on the resistance of sugarcane to *X. albilineans*.

## Conclusions

Infection with *Xanthomonas albilineans* can have destructive consequences for the vascular system of sugarcane plants. So far, the mechanisms by which sugarcane plants defend themselves against pathogens post inoculation have been unclear. Therefore, research into and cloning of resistance genes and breeding resistant varieties should provide effective methods for preventing infection with *X. albilineans*. The present study compared transcriptome profiles in the sugarcane cultivars SES208 and LA Purple before and after *X. albilineans* infection, revealing the apparent activation of an immune response to *X. albilineans* infection in SES208 but not in LA Purple. Genes showing changes in expression during *X. albilineans* infection were annotated with functions including plant-pathogen interactions, MAPK signaling, plant hormone signal transduction, and flavonoid and phenylpropanoid biosynthesis. The expression of many genes often associated with activated disease resistance decreased sharply after infection with *X. albilineans* in SES208, suggesting that its defense mechanisms are worthy of further study. Further, the involvement of putative core resistance genes that exhibit ASE was observed less frequently than that of multi-allelic genes with balanced allele expression, suggesting that the expression of genes with balanced allelic expression might allow synergistic maintenance of this resistance phenotype, especially as the existence of genes exhibiting ASE in *S. saccharum* appears to be a largely genotype-specific phenomenon.

In conclusion, the results of the present study provide useful knowledge for the improvement of resistance to *X. albilineans* in sugarcane and provide new insights into sugarcane responses against *X. albilineans* infection.

## Methods

### Sample collection and *X. albilineans* inoculation

For the present study, seedings of the sugarcane cultivars SES208 and LA Purple were grown in a standard greenhouse at the Center for Genomics and Biotechnology in Fujian Province with a 16-h light (28 °C) and 8-h dark (28 °C) cycle. A single colony of *X. albilineans* strain Xa-FJ1 was suspended in 1 mL of XAS liquid medium [91] and cultured at 28 °C with constant shaking at 200 rpm for 48 h. Then we added 1 μL of the latter culture to 40 mL of fresh XAS liquid medium and incubated at 28 °C for approximately 10 h. The bacterial suspension was diluted to 10^8^ CFU/mL for plant inoculation. The leaves of twenty-day-old seedlings of SES208 and LA Purple with 3–5 leaves were inoculated with *X. albilineans* by cutting the leaf blades at mid-length with sterile scissors dipped in the bacterial suspension. Five control plants were treated in a similar way, but the bacterial suspension was replaced by sterile liquid medium XAS [29]. Leaf samples were collected from the two cultivars at 0 h post inoculation (hpi) hpi (named AP0 hpi and LA0 hpi), 24 hpi (named AP24 hpi and LA24 hpi), 48 hpi (named AP48 hpi and LA48 hpi) and 72 hpi (named AP72 hpi and LA72 hpi). Three biological and three technical replicates were collected for all treatments, immediately frozen in liquid nitrogen, and then stored at − 80 °C prior to RNA extraction.

### RNA extraction and library construction

Total RNA was extracted using the TRIzol® kit (Invitrogen, Carlsbad, CA, USA). The integrity and quality of RNA were assessed using 1.0% agar gel electrophoresis and the NanoDrop 2000 (Thermo Fisher Scientific, USA). An NEBNext® Ultra™ RNA Library Prep Kit for Illumina® was used to construct the RNA-Seq libraries. The sample library concentrations were evaluated using the Qubit® 2.0 fluorimeter with the Qubit dsDNA HS Assay Kit before sending to Novogene (City, Country) for sequencing on the Illumina NovaSeq 6000 System.

### Differential gene expression analysis, weighted correlation network analysis (WGCNA), and function enrichment analysis

The assembly and annotation of the sugarcane AP85-441 genome was downloaded from Ray Ming’s laboratory at (http://www.life.illinois.edu/ming/downloads/Spontaneum_genome/) [92]. We conducted quantitative analysis of RNA-Seq data using Trinity Transcript Quantification. For detailed methods, please refer to the website: https://github.com/trinityrnaseq/trinityranseq/wiki/Trinity-Transcript-Quantification. The default comparison software Bowtie2 [93] and then RSEM [94] were used to calculate TPM (Transcripts Per Kilobase Million) values, and genes with an adjusted *p*-value < 0.001 (|log_2_(fold change)| ≥ 2) found using edgeR [95] were considered as DEGs.

Differentially expressed TFs were predicted using the PlantTFDB website (http://planttfdb.cbi.pku.edu.cn/prediction.php). Then WGCNA analysis was performed using the WGCNA (v1.70-3, https://horvath.genetics.ucla.edu/html/CoexpressionNetwork/Rpackages/WGCNA/) package to find clusters (modules) of genes with highly correlated expression [96].

GO (Gene ontology) enrichment analysis [97] and KEGG (Kyoto Encyclopedia of Genes and Genomes) pathway enrichment analysis [98] of the DEGs was performed using the EggNOG (v5.0) Database (http://eggnog5.embl.de/#/app/home) and OmicShare online tools (https://www.omicshare.com/).

### Quantitative real-time PCR analysis

Each cDNA sample was synthesized using a Prime Script RT Reagent Kit (TaKaRa) and diluted with nuclease-free water to a concentration of 200 ng/μl. Primers to amplify genes chosen for quantitative real-time PCR (qRT-PCR) were designed by IDT (https://sg.idtdna.com/pages) (Additional file 8). Each qRT-PCR was conducted in a 12.5 μl reaction system with the following components: 6.25 μL *SYBR Premix Ex Taq*II (2 ×), 0.5 μL 0.4 μM forward primer, 0.5 μL 0.4 μM reverse primer, 0.5 μL diluted cDNA, and 4.75 μL ddH2O. Each qRT-PCR amplification was conducted on a Bio-Rad CFX96 Real Time PCR System (Bio-Rad, USA) with the following thermal cycling procedure: 95 °C for 3 min, 40 cycles of 95 °C for 10 s, 55 °C for 30 s. The temperature was increased by 0.5 °C every 5 s and the melting curve was generated from 65 °C to 95 °C. The transcript expression of each gene we analyzed was detected in three biological and three technical replications. The eukaryotic *elongation factor 1a* (*eEF-1a*) gene was used as an internal control for normalizing the expression data [99]. Relative gene expression was calculated using the 2^−∆∆CT^ method [100].

## Supplementary Information

**Additional file 1. **Gene Ontology (GO) analysis of DEGs in SES208 and LA Purple infected by *X. albilineans*.

**Additional file 2. **Number of DEGs in major KEGG pathways in MEblack and MEdarkolivegreen module after inoculation with *Xanthomonas albilineans*.

**Additional file 3.** Proportion of alleles in differential genes.

**Additional file 4. **Heatmaps of differentially expressed alleles involved in MAPK signaling pathway, plant-pathogen interaction, plant hormone signal transduction, phenylpropanoid biosynthesis, phenylalanine metabolism, starch and sucrose metabolism, flavonoid biosynthesis and terpenoid-quinone biosynthesis. The expression value was calculated by relative expression of genes (log2 Fold Change from − 2 to + 2). SES: SES208; LA: LA Purple. 0 h: uninfected plants; 24 h, 48 h,and 72 h represent 24, 48 and 72 hours post inoculation of sugarcane with *X. albilineans*, respectively.

**Additional file 5. **Heatmaps of dispersely and tandem duplicated DEGs involved in MAPK signaling pathway, plant-pathogen interaction, plant hormone signal transduction, phenylpropanoid biosynthesis, starch and sucrose metabolism, flavonoid biosynthesis and terpenoid-quinone biosynthesis. The expression value was calculated by relative expression of genes (log2 Fold Change from − 2 to + 2). SES: SES208; LA: LA Purple. 0 h: uninfected plants; 24 h, 48 h,and 72 h represent 24, 48 and 72 hours post inoculation of sugarcane with *X. albilineans*, respectively.

**Additional file 6. **Heatmaps of DEGs involved in phenylpropanoid biosynthesis and starch and sucrose metabolism. The expression value was calculated by relative expression of genes (log2 Fold Change from − 2 to + 2). SES: SES208; LA: LA Purple. 0 h: uninfected plants; 24 h, 48 h,and 72 h represent 24, 48 and 72 hours post inoculation of sugarcane with *X. albilineans*, respectively.

**Additional file 7. **Significant differentially expressed genes (DEGs) information involved in sugarcane response to *X. albilineans* infection.

**Additional file 8.** Primes sequences used in qRT-PCR.

## Data Availability

The raw sequencing reads of transcriptome data have been deposited to the NCBI Sequence Read Archive (SRA) as Bioproject PRJNA893415 (https://www.ncbi.nlm.nih.gov/bioproject/PRJNA893415).

## Abbreviations

DEGs: differentially expressed genes
TF: transcription factor
ASE: Allele-specific expression
LA: Louisiana
HR: hypersensitive response
PTI: PAMP-triggered immunity
PAMPs: pathogen-associated molecular patterns
ETI: effector-triggered immunity
MAPK: mitogen-activated protein kinase
SA: salicylic acid
ABA: abscisic acid
JA: jasmonic acid
ET: ethylene
SAR: systemic acquired resistance
TMV: tobacco mosaic virus
ISR: induced systemic resistance
WGCNA: weighted gene correlation network analysis
hpi: hours post inoculation
PAL: phenylalanine ammonia lyase
4CL: 4-coumarate:CoA ligase
F5H: ferulate-5-hydroxylase
GN4: glucan endo-1,3-beta-glucosidase 4
AMY: alpha-amylase
INV: beta-fructofuranosidase
ISA: isoamylase
glgA: starch synthase
glgC: glucose-1-phosphate adenylyltransferase
GYG1: glycogenin
E2.4.1.34: 1,3-beta-glucan synthase
PYG: glycogen phosphorylase
HK: hexokinase
PRRs: pattern recognition receptors
FLS2: FLAGELLIN SENSING2
RBOHs: respiratory burst oxidase homologues
CAT1/APX1: catalase1/ascorbate peroxidase1
CNGCs: cyclic nucleotide gated channels
RIN: ethylene receptor ripening inhibitor
RPM1: RESISTANCE TO *P. SYRINGAE* PV MACULICOLA 1
RPS2: RIBOSOMAL PROTEIN S2
KCS: 3-ketoacyl-CoA synthase
NPR1: NONEXPRESSER OF PR GENES 1
NPR3: NPR1-LIKE PROTEIN 3
TGA: transglutaminase
PR1: pathogenesis-related protein 1
JAZ: jasmonate ZIM domain
RAN1: response to antagonist 1
ETR: ethylene receptor
CTR1: CONSTITUTIVE TRIPLE RESPONSE 1
EBF1: EIN3-BINDING F BOX PROTEIN 1
EIN3: ETHYLENE-INSENSITIVE3
ERF: ETHYLENE RESPONSE FACTOR
PP2C: type 2C protein phosphatases
SnRK2: class III SNF-1-related protein kinase 2
ABF: abscisic acid-responsive transcription factor
CDPKs: Calcium-dependent protein kinases
LRR: leucine-rich repeat
CHS: chalcone synthase
ANR: anthocyanidin reductase
CYP98A/C3’H: 5-O-(4-coumaroyl)-D-quinate 3′-monooxygenase
CYP75B1: flavonoid 3′-monooxygenase
HPD: 4-hydroxyphenylpyruvate dioxygenase
HPT: homogentisate phytyltransferase
TAT: tyrosine aminotransferase
TPS6: TERPENE SYNTHASE 6
TPM: Transcripts Per Kilobase Million
GO: Gene Ontology.

## References

[1] Botha FC, Moore PH (2013). biomass and bioenergy. In: Sugarcane: Physiology, Biochemistry, and Functional Biology.

[2] Chandra A (2011). Physio-biochemical and molecular approaches to enhance sucrose content in sugarcane: Indian initiatives. Sugar Tech.

[3] Aguiar A, Milessi TS, Mulinari DR, Lopes MS, da Costa SM, Candido RG (2021). Sugarcane straw as a potential second generation feedstock for biorefinery and white biotechnology applications. Biomass Bioenergy.

[4] Zhang J, Zhang X, Tang H, Zhang Q, Hua X, Ma X, Zhu F, Jones T, Zhu X, Bowers J (2018). Allele-defined genome of the autopolyploid sugarcane Saccharum spontaneum L. Nat Genet.

[5] D'Hont A, Grivet L, Feldmann P, Rao S, Berding N, Glaszmann JC (1996). Characterisation of the double genome structure of modern sugarcane cultivars (Saccharum spp.) by molecular cytogenetics. Mol Gen Genet.

[6] O'Hara I, Mundree S (2016). Sugarcane-based biofuels and bioproducts.

[7] Dillon SL, Shapter FM, Henry RJ, Cordeiro G, Izquierdo L, Lee LS (2007). Domestication to crop improvement: genetic resources for Sorghum and saccharum (Andropogoneae). Ann Bot-London.

[8] Brandes EW, B. Sartoris G: sugarcane: its origin and improvement. Yearb US Dep Agric. 1936:561–624.

[9] Rott P, Bailey RA, Comstock JC, Croft BJ, Girard J (2000). Saumtally AS: a guide to sugarcane diseases. D-CAS 1.2: a guide to sugarcane diseases. D-CAS.

[10] Mensi I, Vernerey MS, Gargani D, Nicole M, Rott P. Breaking dogmas: the plant vascular pathogen Xanthomonas albilineans is able to invade non-vascular tissues despite its reduced genome. Open Biol. 2014;4(2):130116. 10.1098/rsob.130116 PMC3938051 24522883

[11] Hashimi SM, Wall MK, Smith AB, Maxwell A, Birch RG (2007). The phytotoxin albicidin is a novel inhibitor of DNA gyrase. Antimicrob Agents Ch.

[12] Johansson ON, Nilsson AK, Gustavsson MB, Backhaus T, Andersson MX, Ellerstrom M (2015). A quick and robust method for quantification of the hypersensitive response in plants. PeerJ.

[13] Ding P, Ding Y (2020). Stories of salicylic acid: a plant defense hormone. Trends Plant Sci.

[14] Han GZ (2019). Origin and evolution of the plant immune system. New Phytol.

[15] Jones JD, Dangl JL (2006). The plant immune system. Nature.

[16] Chisholm ST, Coaker G, Day B, Staskawicz BJ (2006). Host-microbe interactions: shaping the evolution of the plant immune response. Cell.

[17] Asai T, Tena G, Plotnikova J, Willmann MR, Chiu WL, Gomez-Gomez L, Boller T, Ausubel FM, Sheen J (2002). MAP kinase signalling cascade in Arabidopsis innate immunity. Nature.

[18] Torres MA, Dangl JL, Jones JD (2002). Arabidopsis gp91phox homologues AtrbohD and AtrbohF are required for accumulation of reactive oxygen intermediates in the plant defense response. Proc Natl Acad Sci U S A.

[19] Yu X, Feng BM, He P, Shan LB (2017). From Chaos to harmony: responses and signaling upon microbial pattern recognition. Annu Rev Phytopathol.

[20] Yuan M, Ngou BPM, Ding P, Xin XF (2021). PTI-ETI crosstalk: an integrative view of plant immunity. Curr Opin Plant Biol.

[21] White RF (1979). Acetylsalicylic acid (aspirin) induces resistance to tobacco mosaic virus in tobacco. Virology.

[22] Choudhary DK, Prakash A, Johri BN (2007). Induced systemic resistance (ISR) in plants: mechanism of action. Indian J Microbiol.

[23] Piasecka A, Jedrzejczak-Rey N, Bednarek P (2015). Secondary metabolites in plant innate immunity: conserved function of divergent chemicals. New Phytol.

[24] Zaynab M, Fatima M, Abbas S, Sharif Y, Umair M, Zafar MH, Bahadar K (2018). Role of secondary metabolites in plant defense against pathogens. Microb Pathog.

[25] Zhang F, Huang LY, Zhang F, Ali J, Cruz CV, Zhuo DL, et al. Comparative transcriptome profiling of a rice line carrying Xa39 and its parents triggered by Xanthomonas oryzae pv. Oryzae provides novel insights into the broad-spectrum hypersensitive response. BMC Genomics. 2015;16(1):111. 10.1186/s12864-015-1329-3 PMC4349310 25765449

[26] Tariq R, Wang C, Qin T, Xu F, Tang Y, Gao Y, et al. Comparative transcriptome profiling of Rice near-isogenic line carrying Xa23 under infection of Xanthomonas oryzae pv. Oryzae. Int J Mol Sci. 2018;19(3):717. 10.3390/ijms19030717 PMC5877578 29498672

[27] Meng JY, Ntambo MS, Luo LM, Huang MT, Fu HY, Gao SJ. Molecular identification of Xanthomonas albilineans infecting elephant grass (Pennisetum purpureum) in China. Crop Prot. 2019;124:353.

[28] Garcia-Seco D, Chiapello M, Bracale M, Pesce C, Bagnaresi P, Dubois E, et al. Transcriptome and proteome analysis reveal new insight into proximal and distal responses of wheat to foliar infection by Xanthomonas translucens. Sci Rep-Uk. 2017;7(1):10157. 10.1038/s41598-017-10568-8 PMC5579275 28860643

[29] Ntambo MS, Meng JY, Rott PC, Henry RJ, Zhang HL, Gao SJ (2019). Comparative transcriptome profiling of resistant and susceptible sugarcane cultivars in response to infection by Xanthomonas albilineans. Int J Mol Sci.

[30] Meng JY, Ntambo MS, Rott PC, Fu HY, Huang MT, Zhang HL, Gao SJ (2020). Identification of differentially expressed proteins in sugarcane in response to infection by Xanthomonas albilineans using iTRAQ quantitative proteomics. Microorganisms.

[31] Wang HB, Xiao NY, Wang YJ, Guo JL, Zhang JS (2020). Establishment of a qualitative PCR assay for the detection of Xanthomonas albilineans (Ashby) Dowson in sugarcane. Crop Prot.

[32] Gaur U, Li K, Mei S, Liu G (2013). Research progress in allele-specific expression and its regulatory mechanisms. J Appl Genet.

[33] Werck-Reichhart D (1995). Cytochromes P450 in phenylpropanoid metabolism. Drug Metabol Drug Interact.

[34] Nelson D, Werck-Reichhart D (2011). A P450-centric view of plant evolution. Plant J.

[35] Torres MA, Onouchi H, Hamada S, Machida C, Hammond-Kosack KE, Jones JD (1998). Six Arabidopsis thaliana homologues of the human respiratory burst oxidase (gp91phox). Plant J.

[36] Noirot E, Der C, Lherminier J, Robert F, Moricova P, Kieu K, Leborgne-Castel N, Simon-Plas F, Bouhidel K (2014). Dynamic changes in the subcellular distribution of the tobacco ROS-producing enzyme RBOHD in response to the oomycete elicitor cryptogein. J Exp Bot.

[37] Alazem M, Lin NS (2015). Roles of plant hormones in the regulation of host-virus interactions. Mol Plant Pathol.

[38] Pauwels L, Goossens A (2011). The JAZ proteins: a crucial interface in the jasmonate signaling cascade. Plant Cell.

[39] Binder BM (2020). Ethylene signaling in plants. J Biol Chem.

[40] Dittrich M, Mueller HM, Bauer H, Peirats-Llobet M, Rodriguez PL, Geilfus CM, Carpentier SC, Al Rasheid KAS, Kollist H, Merilo E (2019). The role of Arabidopsis ABA receptors from the PYR/PYL/RCAR family in stomatal acclimation and closure signal integration. Nat Plants.

[41] Comai L (2000). Genetic and epigenetic interactions in allopolyploid plants. Plant Mol Biol.

[42] Renny-Byfield S, Wendel JF (2014). Doubling down on genomes: polyploidy and crop plants. Am J Bot.

[43] Yant L, Bomblies K (2015). Genome management and mismanagement--cell-level opportunities and challenges of whole-genome duplication. Genes Dev.

[44] Feldman M, Levy AA (2009). Genome evolution in allopolyploid wheat--a revolutionary reprogramming followed by gradual changes. J Genet Genomics.

[45] Chen ZJ, Ni Z (2006). Mechanisms of genomic rearrangements and gene expression changes in plant polyploids. Bioessays.

[46] Correr FH, Furtado A, Garcia AAF, Henry RJ, Margarido GRA (2022). Allele expression biases in mixed-ploid sugarcane accessions. Sci Rep.

[47] Powell JJ, Fitzgerald TL, Stiller J, Berkman PJ, Gardiner DM, Manners JM, Henry RJ, Kazan K (2017). The defence-associated transcriptome of hexaploid wheat displays homoeolog expression and induction bias. Plant Biotechnol J.

[48] Valliyodan B, Nguyen HT (2006). Understanding regulatory networks and engineering for enhanced drought tolerance in plants. Curr Opin Plant Biol.

[49] Hobert O (2008). Gene regulation by transcription factors and microRNAs. Science.

[50] Liu YL, Schiff M, Dinesh-Kumar SP (2004). Involvement of MEK1 MAPKK, NTF6 MAPK, WRKY/MYB transcription factors, COI1 and CTR1 in N-mediated resistance to tobacco mosaic virus. Plant J.

[51] Bu Q, Jiang H, Li CB, Zhai Q, Zhang J, Wu X, Sun J, Xie Q, Li C (2008). Role of the Arabidopsis thaliana NAC transcription factors ANAC019 and ANAC055 in regulating jasmonic acid-signaled defense responses. Cell Res.

[52] Vannini C, Iriti M, Bracale M, Locatelli F, Faoro F, Croce P, Pirona R, Di Maro A, Coraggio I, Genga A (2006). The ectopic expression of the rice Osmyb4 gene in Arabidopsis increases tolerance to abiotic, environmental and biotic stresses. Physiol Mol Plant P.

[53] Huang PY, Zhang JS, Jiang BE, Chan C, Yu JH, Lu YP, Chung KM, Zimmerli L (2019). NINJA-associated ERF19 negatively regulates Arabidopsis pattern-triggered immunity. J Exp Bot.

[54] Cheng Q, Dong LD, Gao TJ, Liu TF, Li NH, Wang L, Chang X, Wu JJ, Xu PF, Zhang SZ (2018). The bHLH transcription factor GmPIB1 facilitates resistance to Phytophthora sojae in Glycine max. J Exp Bot.

[55] Fan M, Bai MY, Kim JG, Wang TN, Oh E, Chen L, Park CH, Son SH, Kim SK, Mudgett MB (2014). The bHLH transcription factor HBI1 mediates the trade-off between growth and pathogen-associated molecular pattern-triggered immunity in Arabidopsis. Plant Cell.

[56] Nejat N, Mantri N (2017). Plant immune system: crosstalk between responses to biotic and abiotic stresses the missing link in understanding plant Defence. Curr Issues Mol Biol.

[57] Wang D, Liang X, Bao Y, Yang S, Zhang X, Yu H, Zhang Q, Xu G, Feng X, Dou D (2020). A malectin-like receptor kinase regulates cell death and pattern-triggered immunity in soybean. EMBO Rep.

[58] Zou Y, Wang S, Zhou Y, Bai J, Huang G, Liu X, Zhang Y, Tang D, Lu D (2018). Transcriptional regulation of the immune receptor FLS2 controls the ontogeny of plant innate immunity. Plant Cell.

[59] Davletova S, Rizhsky L, Liang HJ, Zhong SQ, Oliver DJ, Coutu J, Shulaev V, Schlauch K, Mittler R (2005). Cytosolic ascorbate peroxidase 1 is a central component of the reactive oxygen gene network of Arabidopsis. Plant Cell.

[60] Gaffney T, Friedrich L, Vernooij B, Negrotto D, Nye G, Uknes S, Ward E, Kessmann H, Ryals J (1993). Requirement of salicylic acid for the induction of systemic acquired resistance. Science.

[61] Chen Z, Silva H, Klessig DF (1993). Active oxygen species in the induction of plant systemic acquired resistance by salicylic acid. Science.

[62] Tang DZ, Wang GX, Zhou JM (2017). Receptor kinases in plant-pathogen interactions: more than pattern recognition. Plant Cell.

[63] Liu B, Li JF, Ao Y, Qu J, Li Z, Su J, Zhang Y, Liu J, Feng D, Qi K (2012). Lysin motif-containing proteins LYP4 and LYP6 play dual roles in peptidoglycan and chitin perception in rice innate immunity. Plant Cell.

[64] Hetherington AM, Brownlee C (2004). The generation of Ca2+ signals in plants. Annu Rev Plant Biol.

[65] Xu W, Huang W. Calcium-dependent protein kinases in Phytohormone signaling pathways. Int J Mol Sci. 2017;18(11):2436. 10.3390/ijms18112436 PMC5713403 29156607

[66] Heo WD, Lee SH, Kim MC, Kim JC, Chung WS, Chun HJ, Lee KJ, Park CY, Park HC, Choi JY (1999). Involvement of specific calmodulin isoforms in salicylic acid-independent activation of plant disease resistance responses. Proc Natl Acad Sci U S A.

[67] Reddy ASN, Ali GS, Celesnik H, Day IS (2011). Coping with stresses: roles of calcium- and calcium/calmodulin-regulated gene expression. Plant Cell.

[68] Mackey D, Holt BF, Wiig A, Dangl JL (2002). RIN4 interacts with pseudomonas syringae type III effector molecules and is required for RPM1-mediated resistance in Arabidopsis. Cell.

[69] Kunkel BN, Bent AF, Dahlbeck D, Innes RW, Staskawicz BJ (1993). RPS2, an Arabidopsis disease resistance locus specifying recognition of pseudomonas syringae strains expressing the avirulence gene avrRpt2. Plant Cell.

[70] Canonne J, Marino D, Jauneau A, Pouzet C, Briere C, Roby D, Rivas S (2011). The Xanthomonas type III effector XopD targets the Arabidopsis transcription factor MYB30 to suppress plant defense. Plant Cell.

[71] Johnson C, Boden E, Arias J (2003). Salicylic acid and NPR1 induce the recruitment of trans-activating TGA factors to a defense gene promoter in Arabidopsis. Plant Cell.

[72] Liu LJ, Sonbol FM, Huot B, Gu YN, Withers J, Mwimba M, et al. Salicylic acid receptors activate jasmonic acid signalling through a non-canonical pathway to promote effector-triggered immunity. Nat Commun. 2016;7:13099. 10.1038/ncomms13099 PMC5062614 27725643

[73] Dombrecht B, Xue GP, Sprague SJ, Kirkegaard JA, Ross JJ, Reid JB, Fitt GP, Sewelam N, Schenk PM, Manners JM (2007). MYC2 differentially modulates diverse jasmonate-dependent functions in Arabidopsis. Plant Cell.

[74] Mandadi KK, Scholthof KB (2013). Plant immune responses against viruses: how does a virus cause disease?. Plant Cell.

[75] Adie BAT, Perez-Perez J, Perez-Perez MM, Godoy M, Sanchez-Serrano JJ, Schmelz EA, Solano R (2007). ABA is an essential signal for plant resistance to pathogens affecting JA biosynthesis and the activation of defenses in Arabidopsis. Plant Cell.

[76] Song W, Ma X, Tan H, Zhou J (2011). Abscisic acid enhances resistance to Alternaria solani in tomato seedlings. Plant Physiol Biochem.

[77] de Zelicourt A, Colcombet J, Hirt H (2016). The role of MAPK modules and ABA during abiotic stress signaling. Trends Plant Sci.

[78] Bellincampi D, Cervone F, Lionetti V (2014). Plant cell wall dynamics and wall-related susceptibility in plant-pathogen interactions. Front Plant Sci.

[79] Li J, Chen M, Fan T, Mu X, Gao J, Wang Y, Jing T, Shi C, Niu H, Zhen S (2022). Underlying mechanism of accelerated cell death and multiple disease resistance in a maize lethal leaf spot 1 allele. J Exp Bot.

[80] Ververidis F, Trantas E, Douglas C, Vollmer G, Kretzschmar G, Panopoulos N (2007). Biotechnology of flavonoids and other phenylpropanoid-derived natural products. Part I: chemical diversity, impacts on plant biology and human health. Biotechnol J.

[81] Long L, Liu J, Gao Y, Xu FC, Zhao JR, Li B, Gao W (2019). Flavonoid accumulation in spontaneous cotton mutant results in red coloration and enhanced disease resistance. Plant Physiol Biochem.

[82] Christensen SA, Huffaker A, Sims J, Hunter CT, Block A, Vaughan MM, Willett D, Romero M, Mylroie JE, Williams WP (2018). Fungal and herbivore elicitation of the novel maize sesquiterpenoid, zealexin A4, is attenuated by elevated CO2. Planta.

[83] Huffaker A, Kaplan F, Vaughan MM, Dafoe NJ, Ni X, Rocca JR, Alborn HT, Teal PE, Schmelz EA (2011). Novel acidic sesquiterpenoids constitute a dominant class of pathogen-induced phytoalexins in maize. Plant Physiol.

[84] Chen F, Ludwiczuk A, Wei G, Chen XL, Crandall-Stotler B, Bowman JL (2018). Terpenoid secondary metabolites in bryophytes: chemical diversity, biosynthesis and biological functions. Crit Rev Plant Sci.

[85] Amini H, Naghavi MR, Shen T, Wang Y, Nasiri J, Khan IA, Fiehn O, Zerbe P, Maloof JN (2019). Tissue-Specific Transcriptome Analysis Reveals Candidate Genes for Terpenoid and Phenylpropanoid Metabolism in the Medicinal Plant Ferula assafoetida. G3 (Bethesda).

[86] Xu Y, Zhu C, Xu C, Sun J, Grierson D, Zhang B, et al. Integration of metabolite profiling and transcriptome analysis reveals genes related to volatile Terpenoid metabolism in finger citron (C. medica var. sarcodactylis). Molecules. 2019;24(14):2564. 10.3390/molecules24142564 PMC6680504 31311090

[87] Zhao J (2015). Flavonoid transport mechanisms: how to go, and with whom. Trends Plant Sci.

[88] Xie Y, Tan H, Ma Z, Huang J (2016). DELLA proteins promote anthocyanin biosynthesis via sequestering MYBL2 and JAZ suppressors of the MYB/bHLH/WD40 complex in Arabidopsis thaliana. Mol Plant.

[89] Mu X, Li J, Dai Z, Xu L, Fan T, Jing T, Chen M, Gou M (2021). Commonly and specifically activated defense responses in maize disease lesion mimic mutants revealed by integrated transcriptomics and metabolomics analysis. Front Plant Sci.

[90] Konig S, Feussner K, Kaever A, Landesfeind M, Thurow C, Karlovsky P, Gatz C, Polle A, Feussner I (2014). Soluble phenylpropanoids are involved in the defense response of Arabidopsis against Verticillium longisporum. New Phytol.

[91] Lin LH, Ntambo MS, Rott PC, Wang QN, Lin YH, Fu HY, Gao SJ (2018). Molecular detection and prevalence of Xanthomonas albilineans, the causal agent of sugarcane leaf scald, in China. Crop Prot.

[92] Zhang JS, Zhang XT, Tang HB, Zhang Q, Hua XT, Ma XK, Zhu F, Jones T, Zhu XG, Bowers J (2018). Allele-defined genome of the autopolyploid sugarcane Saccharum spontaneum L. Nat Genet.

[93] Langmead B, Salzberg SL (2012). Fast gapped-read alignment with bowtie 2. Nat Methods.

[94] Li B, Dewey CN (2011). RSEM: accurate transcript quantification from RNA-Seq data with or without a reference genome. BMC bioinformatics.

[95] Chen Y, Lun AT, Smyth GK (2016). From reads to genes to pathways: differential expression analysis of RNA-Seq experiments using Rsubread and the edgeR quasi-likelihood pipeline. F1000Res.

[96] Langfelder P, Horvath S (2008). WGCNA: an R package for weighted correlation network analysis. BMC bioinformatics.

[97] Young MD, Wakefield MJ, Smyth GK, Oshlack A (2010). Gene ontology analysis for RNA-seq: accounting for selection bias. Genome Biol.

[98] Kanehisa M (2016). KEGG bioinformatics resource for plant genomics and metabolomics. Methods Mol Biol.

[99] Ling H, Wu Q, Guo J, Xu L, Que Y (2014). Comprehensive selection of reference genes for gene expression normalization in sugarcane by real time quantitative rt-PCR. PLoS One.

[100] Livak KJ, Schmittgen TD (2001). Analysis of relative gene expression data using real-time quantitative PCR and the 2(−Delta Delta C(T)) method. Methods.

