# Supplementary material for: Transcriptome analysis of sugarcane reveals rapid defense response of SES208 to Xanthomonas albilineans in early infection
Source: BMC Plant Biol. 2023 Jan 24;23:52. doi: 10.1186/s12870-023-04073-6 (PMC9872421; doi:10.1186/s12870-023-04073-6)

**Additional file 1.** Gene Ontology (GO) analysis of DEGs in SES208 and LA Purple infected by *X. albilineans*.


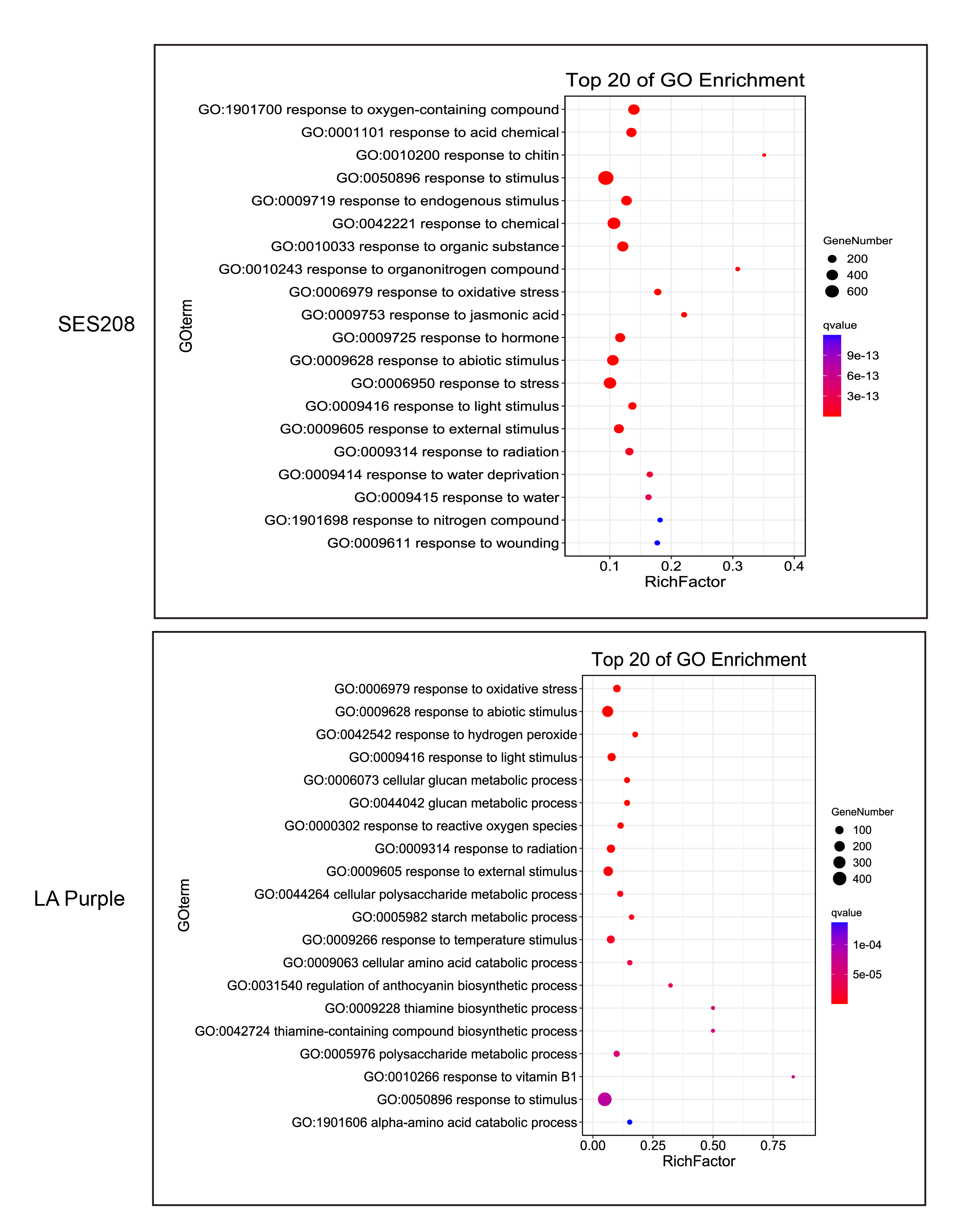

Supplement: Supplementary file 1 — Additional file 1. Gene Ontology (GO) analysis of DEGs in SES208 and LA Purple infected by X. albilineans. [file 12870_2023_4073_MOESM1_ESM.docx]
