# Supplementary figures and images for: Transcriptome analysis of sugarcane reveals rapid defense response of SES208 to Xanthomonas albilineans in early infection
Source: BMC Plant Biol. 2023 Jan 24;23:52. doi: 10.1186/s12870-023-04073-6 (PMC9872421; doi:10.1186/s12870-023-04073-6)

**Additional file 3.** Proportion of alleles in differential genes


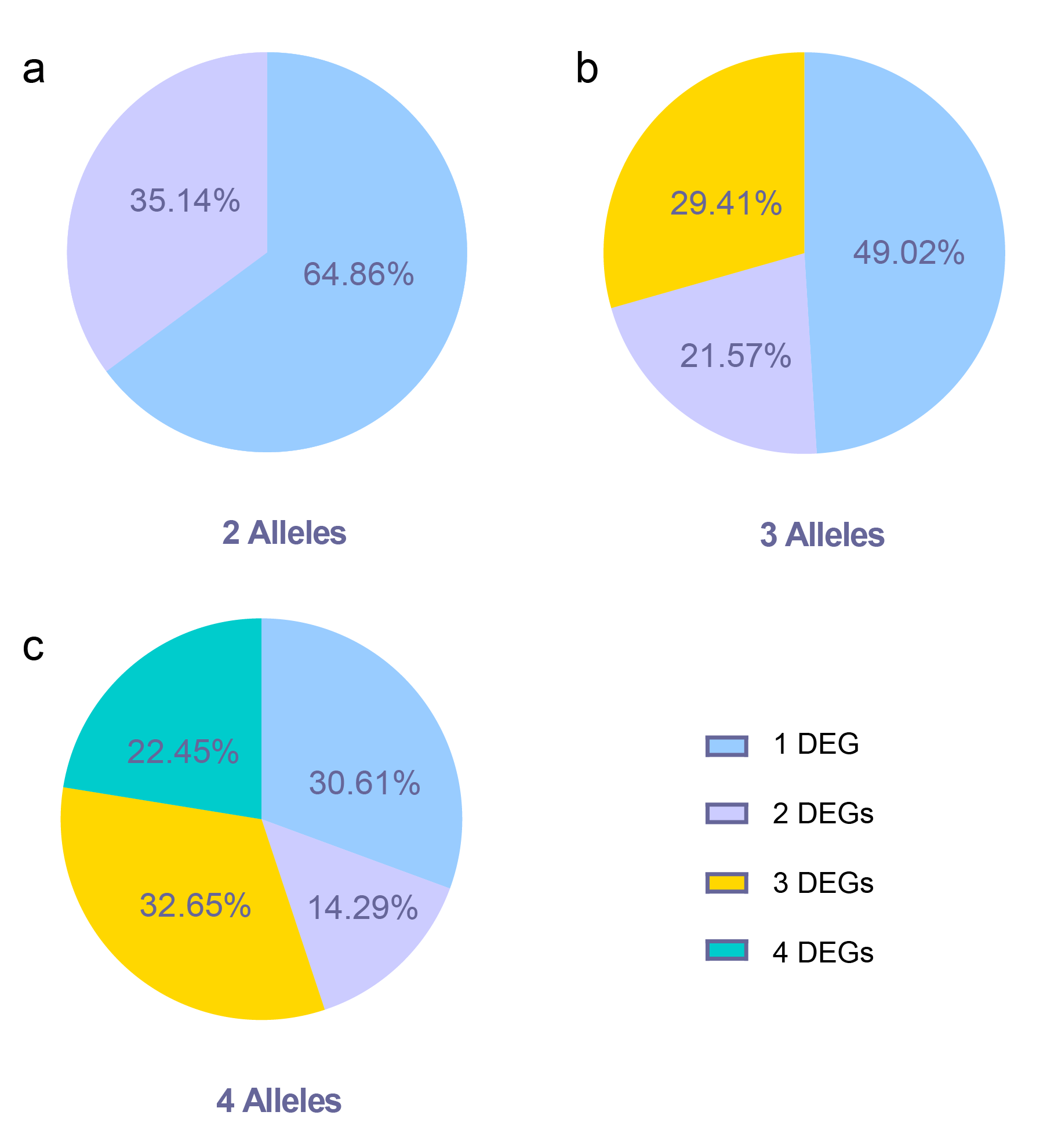

Supplement: Supplementary file 3 — Additional file 3. Proportion of alleles in differential genes. [file 12870_2023_4073_MOESM3_ESM.docx]
