# Supplementary material for: Transcriptome analysis of sugarcane reveals rapid defense response of SES208 to Xanthomonas albilineans in early infection
Source: BMC Plant Biol. 2023 Jan 24;23:52. doi: 10.1186/s12870-023-04073-6 (PMC9872421; doi:10.1186/s12870-023-04073-6)

**Additional file 4.** Heatmaps of differentially expressed alleles involved in MAPK signaling pathway, plant-pathogen interaction, plant hormone signal transduction, phenylpropanoid biosynthesis, phenylalanine metabolism, starch and sucrose metabolism, flavonoid biosynthesis and terpenoid-quinone biosynthesis. The expression value was calculated by relative expression of genes (log2 Fold Change from -2 to +2). SES: SES208; LA: LA Purple. 0h: uninfected plants; 24h, 48h,and 72h represent 24, 48 and 72 hours post inoculation of sugarcane with *X. albilineans*, respectively.


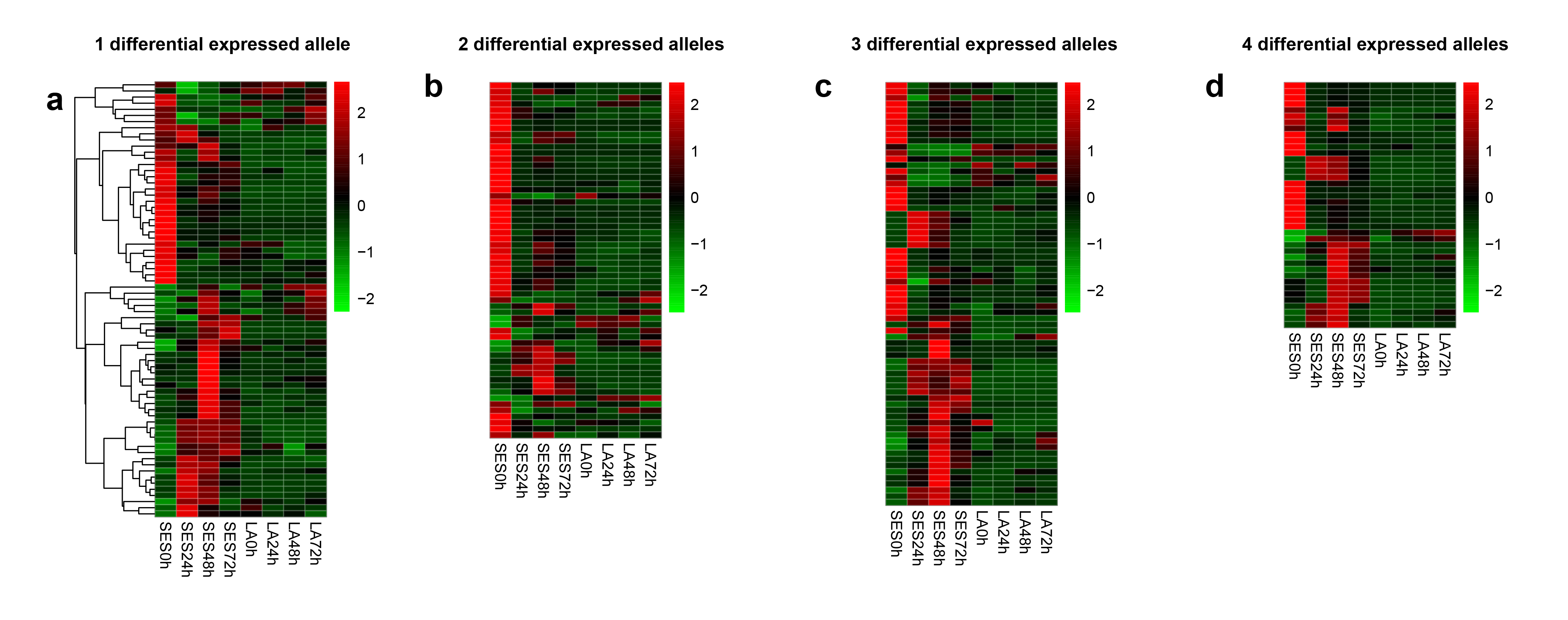

Supplement: Supplementary file 4 — Additional file 4. Heatmaps of differentially expressed alleles involved in MAPK signaling pathway, plant-pathogen interaction, plant hormone signal transduction, phenylpropanoid biosynthesis, phenylalanine metabolism, starch and sucrose metabolism, flavonoid biosynthesis and terpenoid-quinone biosynthesis. The expression value was calculated by relative expression of genes (log2 Fold Change from − 2 to + 2). SES: SES208; LA: LA Purple. 0 h: uninfected plants; 24 h, 48 h,and 72 h represent 24, 48 and 72 hours post inoculation of sugarcane with X. albilineans, respectively. [file 12870_2023_4073_MOESM4_ESM.docx]
