# Supplementary material for: Transcriptome analysis of sugarcane reveals rapid defense response of SES208 to Xanthomonas albilineans in early infection
Source: BMC Plant Biol. 2023 Jan 24;23:52. doi: 10.1186/s12870-023-04073-6 (PMC9872421; doi:10.1186/s12870-023-04073-6)
Supplement: Supplementary file 8 — Additional file 8. Primes sequences used in qRT-PCR. [file 12870_2023_4073_MOESM8_ESM.docx]

**Additional file 8.** Primes sequences used in qRT-PCR

| Target gene | Gene ID | Forward primer (5'-3') | Reverse primer (5'-3') |
| --- | --- | --- | --- |
| *eEF* |  | TTTCACACTTGGAGTGAAGCAGAT | GACTTCCTTCACAATCTCATCATAA |
| *MYC2* | Sspon.01G0015170-1A | GCTGAACCAGCGCTTCTA | GAGCTCGTTGATGTAGGAGATG |
| *WRKY33* | Sspon.03G0029850-1B | CTCCACTCTTCCGCTAACATC | CTTGCCGAGATGCGATCA |
| *CAT1* | Sspon.04G0021320-1A | CCACACCTTCTTCTTCCTCTTC | GAACTTCACGTAGTGGGACTT |
| *EBF1* | Sspon.08G0006270-3P | CGGGTTACTCTTGTCGGTATTC | GCAGAGCAGATATCCTTGATCC |
